# In search of visible-light photoresponsive peptide nucleic acids (PNAs) for reversible control of DNA hybridization

**DOI:** 10.3762/bjoc.15.243

**Published:** 2019-10-22

**Authors:** Lei Zhang, Greta Linden, Olalla Vázquez

**Affiliations:** 1 Fachbereich Chemie, Philipps-Universität Marburg, Hans-Meerwein Straße 4, 35043 Marburg, Germany

**Keywords:** azobenzene, hemithioindigo, peptide nucleic acid (PNA), photoswitch, visible-light irradiation

## Abstract

Photoswitchable oligonucleotides can determine specific biological outcomes by light-induced conformational changes. In particular, artificial probes activated by visible-light irradiation are highly desired in biological applications. Here, we report two novel types of visible-light photoswitchable peptide nucleic acids (PNAs) based on the molecular transducers: hemithioindigo and tetra-*ortho*-fluoroazobenzene. Our study reveals that the tetra-*ortho*-fluoroazobenzene–PNA conjugates have promising properties (fast reversible isomerization, exceptional thermal stability, high isomer conversions and sensitivity to visible-light irradiation) as reversible modulators to control oligonucleotide hybridization in biological contexts. Furthermore, we verified that this switchable modification delivers a slightly different hybridization behavior in the PNA. Thus, both melting experiments and strand-displacement assays showed that in all the cases the *trans*-isomer is the one with superior binding affinities. Alternative versions, inspired by our first compounds here reported, may find applications in different fields such as chemical biology, nanotechnology and materials science.

## Introduction

Light-driven control of oligonucleotide hybridization has demonstrated an enormous potential to regulate on-demand biological responses such as gene expression [1]. There are indeed a number of successful examples based on photocaged strategies [2–5], in which modified nucleic acids interfere irreversibly with gene expression in vitro [6–7] and in vivo [8–12]. Manipulation of gene expression demonstrated therapeutic application – antisense chemistry [13]. Along these lines, photopharmacology [14–15] is an emerging field that highlights the importance of reversible photocontrollable drugs in tomorrow’s medicine, but photoswitchable antisense research in the context of photopharmacology is entirely unexplored. Furthermore, reversible approaches with photoswitches will contribute to a better understanding of biological pathways as they would allow precise reversible spatio-temporal activation/deactivation of the desired targets without causing a permanent knockout. During the last years, the pioneering structural studies of reversible photoregulation of DNA/RNA duplex stability of Asanuma and Komiyama [16–17] have become functional ones, affecting DNA/RNA cleavage [18–20], transcription [21–23], and translation [24–25]. Except a handful of current examples [22,24,26], most of these photoresponsive oligonucleotides are canonical ones where the classical azobenzene is the prominently used photoswitch; although spiropyrans [27], stilbenes [28], diarylethanes [29] and overcrowded alkenes [30] have also been employed. In vivo application demands the development of a new generation of artificial agents to target DNA/RNA-associated processes. These compounds must be able to maintain their specificity and effectivity while still being nuclease resistant, nontoxic and susceptible to light of tissue-penetrating wavelengths. Peptide nucleic acids (PNAs) [31] are synthetic nucleic acid analogues, in which nucleobases are linked to a repeating *N*-(2-aminoethyl)glycine polyamide backbone. The lack of phosphate groups provides them with both higher binding affinities to complementary DNA or RNA sequences and improved mismatch discrimination under physiological conditions than natural ones. Furthermore, PNAs have a straightforward chemical synthesis by Fmoc-based PNA solid-phase synthesis and remarkable stability against nuclease- and protease-mediated degradation [32–33]. In regard to all these beneficial properties of PNA, they may become a promising alternative to overcome the current limitations of the available photoswitchable DNA- and RNA-based systems with potential for in vivo applications too.

**Figure 1:**
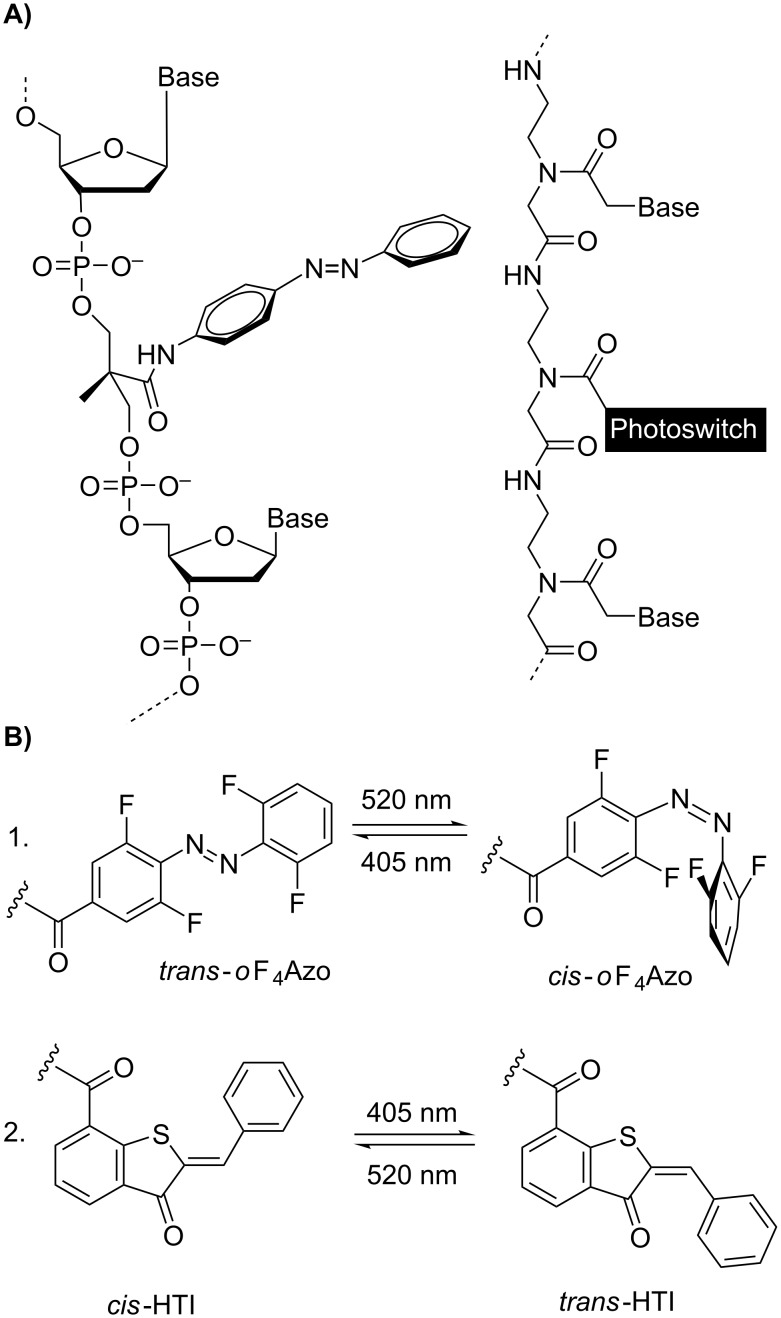
A) Structure of the pioneering azobenzene-modified DNA [16] compared with the photoswitchable PNA structure based on a monomer surrogate. B) Isomerization of the molecular transducers incorporated and studied in this project: 1.: tetra-*ortho*-fluoroazobenze (*o*F_4_Azo) and 2.: hemithioindigo (HTI).

Only very few is known about the reversible hybridization of PNAs upon irradiation [34–36]. Besides these precedents use azobenzene-containing PNA to mainly regulate PNA/DNA triplex helix formation by illumination at low wavelengths (360 nm/425 nm) [35–36]. This effect was successfully exploited for the photocontrol of transcription by T7 RNA polymerase in vitro [36]. In fact, such a result opens new avenues for the investigation of other photoswitchable PNAs and pursuing visible-light modulation. Herein, we report the design of a versatile synthetic platform to derivatize PNAs with different photoswitches (Figure 1), which has never been studied in the context of PNA. After incorporation, their switching capacities and duplex formation were analyzed.

Our group has recently demonstrated that photoresponsive peptides can affect the transcription of genes via inhibition of histone-modifying enzymes [37]. Repression of enzymes is achievable at nucleic acid level too. Therefore, in this project we envision a minimal model based on the previously reported accessible mRNA region of the class I histone deacetylase HDAC-1: 5’-GUGAGCCAAGAAACACUGCCU-3’ to investigate our photoswitchable PNAs [38]. Importantly, HDAC-1 is frequently overexpressed in tumors and particularly, in prostate cancer [39].

## Results and Discussion

Initially, short 12-mer PNA probes (Table 1) complementary to the HDAC-1 mRNA sequence were synthesized since, in general, PNAs are active with shorter sequences than the canonical analogues due to the superior binding abilities [40]. Despite that target specificity may be compromised, 12-mer long sequences are a suitable starting point for our preliminary tests. An overview of all the sequences of this study can be found in Table 1 and Table 2.

**Scheme 1:**
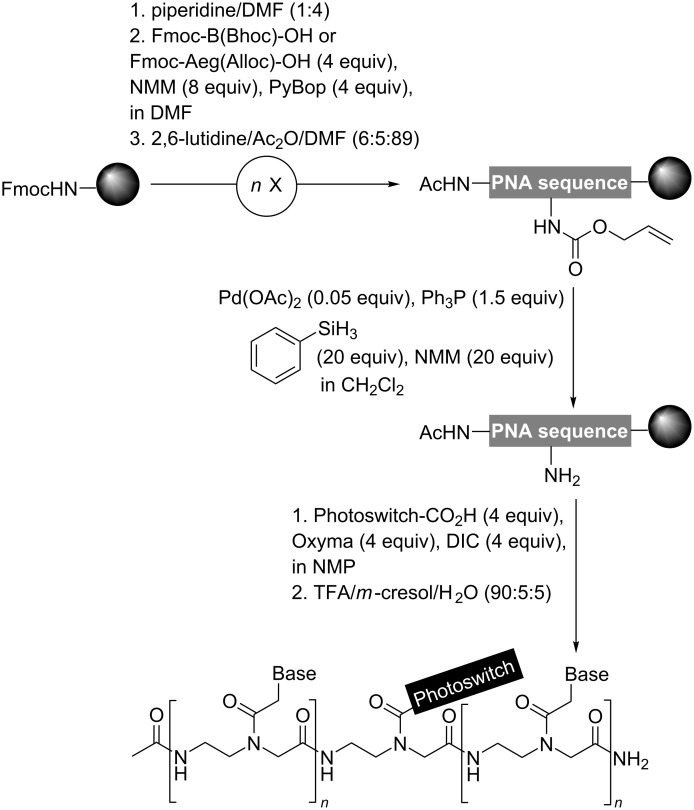
Solid-phase synthesis of photoswitchable PNAs; Aeg = *N*-(2-aminoethyl)glycine, Bhoc = benzhydryloxycarbonyl.

Contrary to the previous synthetic approach of azobenzene-containing PNAs [35–36,41], in which the preformed monomer building block was used, we gained versatility using the divergent linear approach introduced by Seitz (Scheme 1) [42]. This strategy enabled the straightforward access to functionalized PNA via on-resin coupling of the corresponding photoswitch in good yields. Of note, this post-synthetic modification is compatible with base sensitive compounds, which undergo degradation under standard Fmoc deprotection conditions. As it is common for PNAs, our oligomers have an acetylated N-terminus and a C-terminal carboxamide group. After completion of the PNA sequences, the orthogonally protected backbone module [2-(*N*-Alloc)aminoethyl]glycine residue –Aeg(Alloc)– was selectively deprotected in the presence of Pd(OAc)_2_, Ph_3_P, NMM, PhSiH_3_ in CH_2_Cl_2_ for 2 h. Subsequently, carboxy photoswitches were introduced using Oxyma and *N*,*N*’-diisopropylcarbodiimide (DIC) as coupling agent.

We explored two different types of photoswitches (Figure 1B): 1) second generation azobenzenes based on the tetra-*ortho*-fluoroazobenze (*o*F_4_Azo) developed by Hecht [43] and 2) hemithioindigos (HTI) rediscovered by Rück-Braun [44] and Dube [45], which have not been studied in the context of DNA/RNA as molecular transducer yet. Both compounds were compatible with the standard TFA/*m*-cresol/H_2_O (90:5:5) acidolysis yielding the photoswitchable PNA conjugates. These and all further studied PNAs in this work (Table 2) were purified by reversed-phase (RP) HPLC and fully characterized. Analogous compounds lacking the photoswitch were used as controls.

First, the photochromic behavior of the newly synthesized modified PNAs (Table 1, compound **3** and **4**) was investigated and compared with the corresponding photoswitchable PNA monomers (Table 1, compound **1** and **2**).

**Table 1:** Isomerization conversions at the photostationary state (PSS).

Compound	Isomer ratio^a^ [%]	

Ac-Aeg(*o*F_4_Azo)CONH_2_ (**1**)	*trans*	91
	*cis*	90

Ac-Aeg(HTI)CONH_2_ (**2**)	*trans*	50
	*cis*	99

PNA_12_(*o*F_4_Azo) (**3**): Ac-ggcagAeg(*o*F_4_Azo)gtttct-CONH_2_	*trans*	95
	*cis*	82

PNA_12_(HTI) (**4**): Ac-ggcagAeg(HTI)gtttct-CONH_2_	*trans*	47
	*cis*	93

^a^Isomer ratios of a 20 µM solution of the corresponding compound in phosphate buffer (10 mM NaH_2_PO_4_, 150 mM NaCl, pH 7.4) were determined at the isosbestic point (275 nm, for *o*F_4_Azo and HTI) by RP-HPLC; irradiation to obtain the *cis-*isomer at PSS: 520 nm for 10 min and the *trans-*isomer at PSS: 405 nm for 2 min. Mean values derived from two independent experiments; Aeg = *N*-(2-aminoethyl)glycine.

Apart from the expected increase of the band at 260 nm due to the aromatic base moieties within the PNAs, UV–vis spectroscopy of 20 μM solutions in phosphate buffer (10 mM NaH_2_PO_4_, 150 mM NaCl, pH 7.4) confirmed that PNA incorporation did not significantly affect the photochromism (Figures S24, S20, S30 and S33, Supporting Information File 1). This verifies the integrity of the chromophores after the cleavage from the solid support. Among the initial studied photoswitchable PNAs, the PNA_12_(*o*F_4_Azo) (**3**) displayed the most promising properties as reversible modulator of oligonucleotide hybridization. Thus, it displayed the fastest reversible isomerization (≈2 s for *cis* → *trans* and ≈120 s for *trans* → *cis* at these conditions, Figure S24C, Supporting Information File 1) without any signs of photodegradation and photochemical fatigue up to 20 cycles under visible-light irradiation. Regarding the photoconversion ratios between isomers (Table 1), PNA_12_(*o*F_4_Azo) (**3**) had the best ratios. However, the large separation between the n → π* bands of the *trans* and the *cis*-forms (Δλ = 69 nm; Figure S24, Supporting Information File 1) did not lead to the quasi-quantitative conversion for the *cis*-isomer, as for the photoswitchable PNA monomer **1** and the molecular transducer [43,46]. This slightly lower *cis* ratio was also reported in photoswitchable peptides and DNA binders equipped with *o*F_4_Azo [47–48].

**Figure 2:**
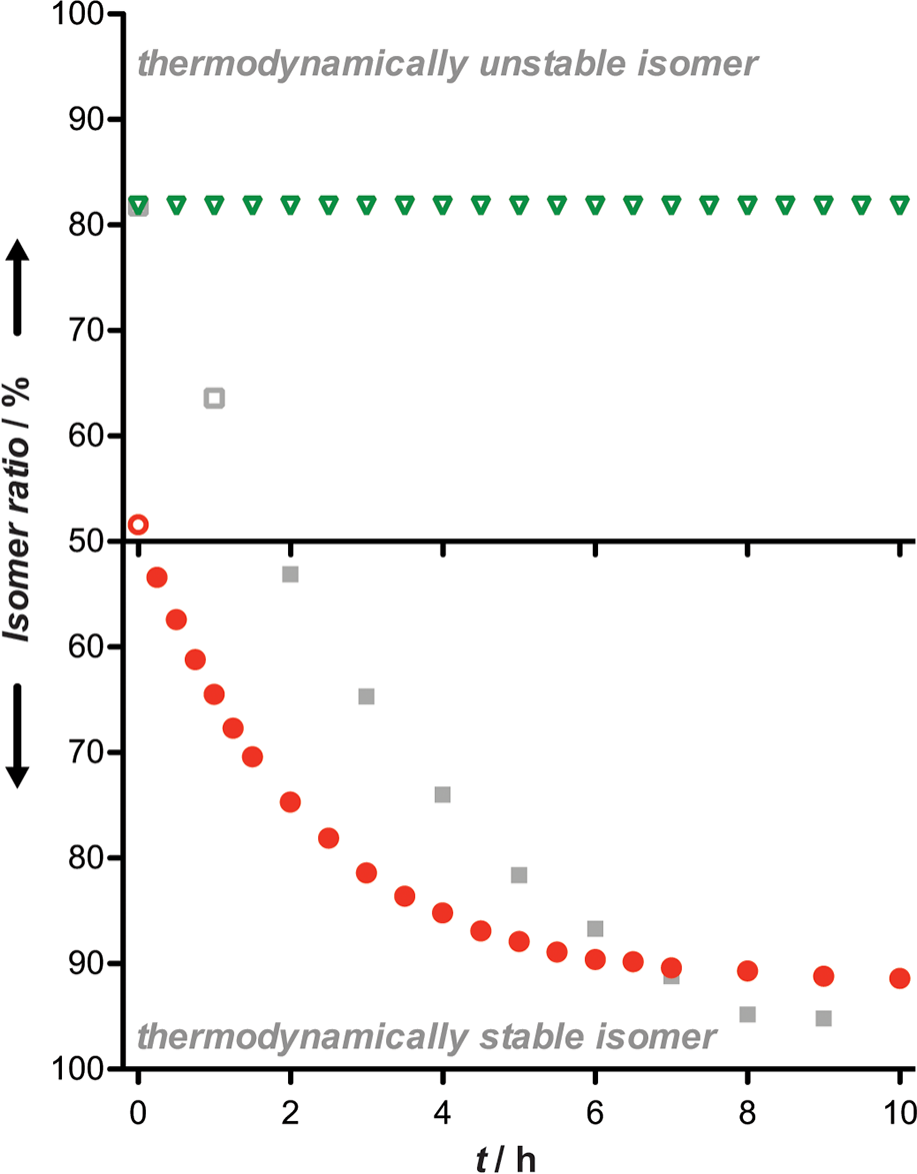
Time-dependent conversion to the thermodynamically stable isomer of PNA_12_(*o*F_4_Azo) (**3**; green triangles) and PNA_12_(HTI) (**4**; red circles). 20 µM solutions of the corresponding compound in phosphate buffer (10 mM NaH_2_PO_4_, 150 mM NaCl, pH 7.4) were irradiated to obtain maximal *cis* (*o*F_4_Azo, 520 nm, 10 min) or *trans* (HTI, 405 nm, 2 min) and stored in the dark. PNA_12_(*o*F_4_Azo) (**3**) was also measured with continuous heating at 90 °C (gray squares). UV–vis spectra were collected at different time points during 10 h. Curves derived were calculate from two independent experiments where the value of the absorbance at 304 nm (for **3**) or at 442 nm (for **4**) was measured.

Regarding stability, the *cis-*PNA_12_(*o*F_4_Azo) (**3**) was stable at least for 24 h at 37 °C, while under the same conditions the thermodynamically unstable isomer of PNA_12_(HTI) (**4**) reverted after 6 h at room temperature in the dark according to UV–vis measurements (Figure 2 and Figure S32, Supporting Information File 1). The thermal relaxation of the *cis-*PNA_12_(*o*F_4_Azo) (**3**) was slow even at high temperatures (Figures S27 and S28, Supporting Information File 1). Furthermore, RP-HPLC chromatograms of its *cis*-form did not show any decomposition under these conditions (Figure S27C, Supporting Information File 1), unlike when the *o*F_4_Azo was grafted onto a pyrrole scaffold [48].

**Figure 3:**
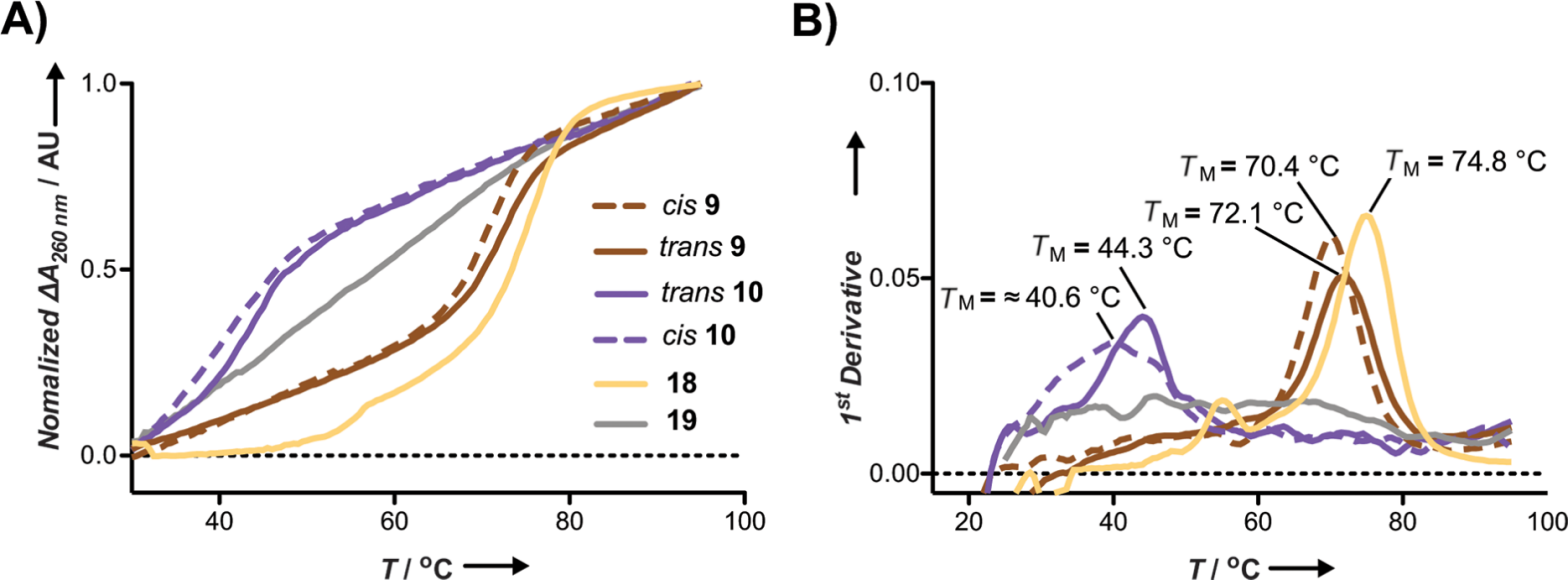
A) Melting curves of a 1 µM duplex solution in phosphate buffer (10 mM NaH_2_PO_4_, 150 mM NaCl, pH 7.4) of the PNA (**18** = yellow solid line; **19** = grey solid line; **9** = brown, *cis* dashed line, *trans* solid line; **10** = purple, *cis* dashed line, *trans* solid line and the complementary ssDNA (5’-GTG AGC CAA GAA ACA CTG CCT-3’)). The melting curves were duplicates from two independent experiments combining 3 cycles of measurements from 20 °C to 95 °C at 260 nm without pre-hybridization. B) Melting temperatures (*T*_M_) obtained by the first derivative of the data in A.

Next, we explored if the inclusion of *o*F_4_Azo as monomer surrogate within the PNA sequence could affect its hybridization to a complementary DNA. For this purpose, we decided to measure thermal melting curves. Beforehand we verified the photostability of *cis*-PNA_12_(*o*F_4_Azo) (**3**) in the temperature ramp from 20 °C to 90 °C by UV–vis spectroscopy. We observed that the *cis*-isomer was always stable during the whole temperature range (Figure S27B, Supporting Information File 1). Remarkably, its time-dependent conversion to the *trans*-PNA_12_(*o*F_4_Azo) (**3**) at 90 °C was surprisingly slow (Figure 2 and Figure S28, Supporting Information File 1) in contrast to the reported *cis*-azobenzene tethered oligonucleotides [49]. Therefore, such unique characteristic allowed the irradiation of the photoswitchable PNA before hybridization (see Supporting Information File 1 for the detailed procedure). Thermal melting profiles showed single sigmoidal transitions (Figure 3 and Figures S37–S45, Supporting Information File 1), which enables the calculation of the melting temperatures (*T*_M_) by the analysis of the first derivative [50]. *T*_M_ values are summarized in Table 2.

**Table 2:** Melting temperatures (*T*_M_) of the duplex between the PNAs and the complementary ssDNA (5’-GTG AGC CAA GAA ACA CTG CCT-3’).

	Sequence	*T*_M_ [°C]	
		*cis*	*trans*

**3**	Ac-ggcag**Aeg(*o*F_4_Azo)**gtttct-CONH_2_	50.5 ± 0.04	50.5 ± 0.02
**5**	Ac-ggcag**Aeg(Ac)**gtttct-CONH_2_	44.3 ± 0.4	

**6**	Ac-*Lys-***Aeg(*o*F_4_Azo)**gcagtgtttcttgg-*Lys*-CONH_2_	70.8 ± 0.3	70.6 ± 0.2
**7**	Ac-*Lys-*gg**Aeg(*o*F_4_Azo)**agtgtttcttgg-*Lys*-CONH_2_	61.4 ± 0.5	62.4 ± 0.4
**8**	Ac-*Lys-*ggcagt**Aeg(*o*F_4_Azo)**tttcttgg-*Lys*-CONH_2_	56.8 ± 0.5	57.1 ± 0.4
**9**	Ac-*Lys*-ggcagtgtttcttg**Aeg(*o*F_4_Azo)**-*Lys*-CONH_2_	70.4 ± 0.8	72.1 ± 0.4
**10**	Ac-*Lys*-ggc**Aeg(*o*F_4_Azo)**gtgtttc**Aeg(*o*F_4_Azo)**tgg-*Lys*-CONH_2_	≈40.6^a^ ± 1.0	44.3 ± 0.8
**11**	Ac-*Lys*-gg**Aeg(*o*F_4_Azo)**agtg**Aeg(*o*F_4_Azo)**ttct**Aeg(*o*F_4_Azo)**gg-*Lys*-CONH_2_	n.c.	n.c.

**12**	Ac-*Lys*-**Aeg(Azo)**-gcagtgtttcttgg-*Lys*-CONH_2_	71.5^b^ ± 0.7	71.5 ± 0.4
**13**	Ac-*Lys*-gg**Aeg(Azo)**agtgtttcttgg-*Lys*-CONH_2_	62.7^b^ ± 0.3	63.0 ± 0.3
**14**	Ac-*Lys*-ggcagt**Aeg(Azo)**tttcttgg-*Lys*-CONH_2_	57.3^b^ ± 0.3	58.0 ± 0.3
**15**	Ac-*Lys*-ggcagtgtttcttg**Aeg(Azo)**-*Lys*-CONH_2_	70.0^b^ ± 0.8	73.2 ± 0.3
**16**	Ac-*Lys*-ggc**Aeg(Azo)**gtgtttc**Aeg(Azo)**tgg-*Lys*-CONH_2_	42.1^b^ ± 0.6	44.5 ± 0.6
**17**	Ac-*Lys*-gg**Aeg(Azo)**agtg**Aeg(Azo)**ttct**Aeg(Azo)**gg-*Lys*-CONH_2_	n.c.	n.c.

**18**	Ac-*Lys*-ggcagtgtttcttgg-*Lys*-CONH_2_	74.8 ± 0.3	
**19**	Ac-*Lys*-tgagtgcgtctgttg-*Lys*-CONH_2_	n.c.	

**20**	Ac-ggcagtgtttct-CONH_2_	64.5^c^	

^a^This value might be approximate according to its 1st derivative (Figure 3B); ^b^PNA and ssDNA (5’-GTG AGC CAA GAA ACA CTG CCT-3’) were isomerized to the *cis*-form after hybridization, following reported procedures [36]; ^c^theoretical value calculated by PNA Bio Tools (http://www.pnabio.com); n.c. = not calculated. Mean values derived from two independent experiments; Aeg = *N*-(2-aminoethyl)glycine.

The incorporation of the visible-light responsive azobenzene (PNA **3**) stabilized the duplex in comparison with the Aeg(Ac)-modified analogue **5**. However, the *T*_M_ of **3** is 14 °C lower than the one calculated for the unmodified PNA analogue (**20**). Unfortunately, no difference between isomers was observed. The potential of the functionality of our compounds could be enhanced by both changing the localization and the number of incorporated photoswitches. To try to maintain the cooperative base pairing, we synthesized longer PNA conjugates (**6**–**11**, **18** and **19**) with flanking lysines, which improved the solubility of the probes. In addition, we compared our probes with the ones that contained the classical unmodified azobenzene moiety (Azo) (PNA **12**–**17**). The lower stability of the unmodified *cis*-Azo forced us to perform the isomerization after duplex hybridization, which resulted in only a constant 36% *cis*-isomer rate. The obtained *T*_M_ values suggested that the localization of the *o*F_4_Azo affects both the duplex stability and isomer differences. Thus, the incorporation of *o*F_4_Azo near the center of the PNA (**7** and **8**) sequence dramatically destabilized the PNA/DNA duplex (Δ*T*_M_ = 18 °C), which agrees with excellent capacity of PNAs for effectively discriminate between single base mismatches. However, when the exchanged base is at either the N- or C-terminus of the PNA (**6** and **9**) the effect is not that dramatic. As reported in the precedent of azobenzene-PNA [51], the C-terminus modification displayed the highest isomer difference but modest, in our case. Furthermore, always when a clear difference between isomers was detected, the duplexes containing the *trans*-*o*F_4_Azo were more stable than those with the *cis*-form. In addition, we tested PNAs with the photoswitch at two (**10**) and three positions (**11**). The *T*_M_ uniformly decreased with the number of *o*F_4_Azo to the point that the probe **11** with three *o*F_4_Azo behaved as the scramble PNA control **19**; this means, it did not form a stable duplex. More interestingly, we could observe an improved *T*_M_ difference between the *trans*- and *cis*-PNA_15_(*o*F_4_Azo)_2_ **10** (up to ≈3.7 °C), which was the best for our system.

Azobenzene (Azo)-containing PNAs behaved similarly to PNA(*o*F_4_Azo) (Table 2), which verified that the fluorine substitution did not affected the binding properties. The differences between isomers were also qualitatively consistent with a slight improvement for the case of the dual-labelled PNA(*o*F_4_Azo) **10**.

**Figure 4:**
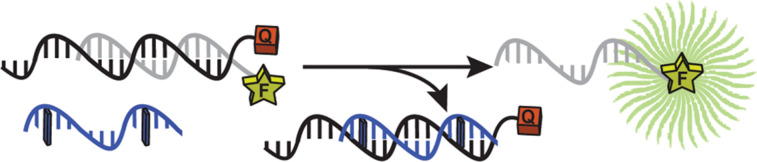
Outline of the displacement assay principle, in which a photoswitchable PNA probe (blue) hybridizes to a complementary quencher-labelled single-stranded (ss) DNA (black) and replaces a fluorescent-labelled ssDNA (grey); F = fluorescein; Q = black hole quencher; blue rectangle = tetra-*ortho*-fluoroazobenzene moiety.

To corroborate our results, we developed a strand-displacement assay using fluorescence as readout. We designed a system based on three molecules (Figure 4): a black hole quencher (BHQ)-labelled single-strand (ss) DNA template, a complementary fluorescein (FAM)-labelled ssDNA and the PNA of interest. In this framework, the PNA could bind the BHQ-ssDNA; thus, its addition to the quenched BHQ/FAM-DNA duplex would trigger an exchange reaction and the release of the FAM-ssDNA. The FAM probe and the PNAs shared the same 15 base-pair sequence (Table S2, Supporting Information File 1). Fluorescence spectroscopy determined the hybridization degree and, in turn, the effect of the photoswitch.

After optimization, we found the following conditions: 0.75 μM quenched double-stranded (ds) DNA, 2 equiv PNA for 8 hours at 37 °C, as the best ones for our assay performance. As expected, the formed FAM/BHQ-dsDNA quenched effectively the fluorescence of FAM-ssDNA (Figure S46, Supporting Information File 1), which was quantitatively restored in the presence of the unmodified PNA **18**. The effect of the scrambled PNA **19** is the opposite, i.e., signal decrease. We determined a slightly lower fluorescence value than in the case of BHQ-ssDNA; this can be probably attributed to the quenching ability of both PNA probes [52]. Along these lines, the quenching ability of *o*F_4_Azo-containing PNA was evaluated (Figure S46, Supporting Information File 1; Table 3) and neglected because of the low impact.

**Table 3:** Normalized increase of fluorescence signal derived from the strand-displacement assays.^a^

Compound	Fluorescence increase at 37 °C [%]		Fluorescence increase at 30 °C [%]	

	15-mer FAM-ssDNA		11-mer FAM-ssDNA	
	*cis*	*trans*	*cis*	*trans*

**6**	36.8 ± 1.2	36.3 ± 1.3	69.9 ± 3.4	75.8 ± 2.6
**7**	43.1 ± 1.7	45.9 ± 1.0	78.1 ± 4.3	82.1 ± 1.2
**8**	44.0 ± 2.0	45.0 ± 1.3	67.0 ± 0.9	72.7 ± 1.9
**9**	46.1 ± 1.3	49.0 ± 1.5	84.7 ± 2.1	82.7 ± 3.5
**10**	n.d.	n.d.	37.8 ± 2.2	46.3 ± 2.2
**11**	n.d.	n.d.	n.d.	n.d.

**18**	98.8 ± 1.4		94.5 ± 3.1	
**19**	17.0 ± 0.4		27.9 ± 3.2	

FAM-ssDNA	100		100	
background^b^	0		0	
BHQ/FAM-dsDNA	24.5 ± 0.3		36.6 ± 0.3	

^a^Percentages calculated according to the measured endpoint (8 h) fluorescence intensity values and considering the FAM-ssDNA intensity as 100%; ^b^it represents the measurement of the PBS buffer: 140 mM NaCl, 10 mM Na_2_HPO_4_, 2.7 mM KCl, 1.8 mM KH_2_PO_4_, pH 8.0. Mean values derived from two independent experiments; n.d. = no displacement observed.

**Figure 5:**
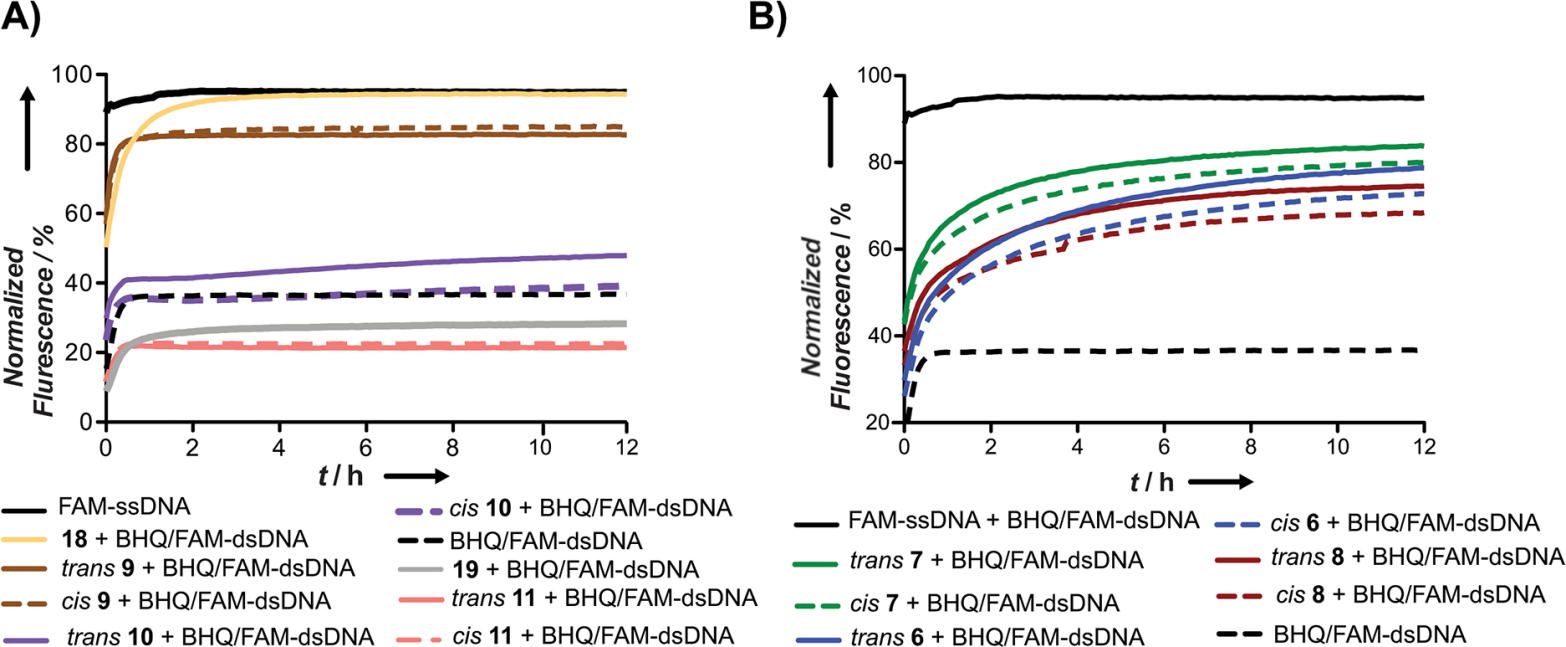
Time-dependent fluorescence signals from two independent experiments at 520 nm of 0.75 μM FAM/BHQ-dsDNA solutions in PBS buffer (140 mM NaCl, 10 mM Na_2_HPO_4_, 2.7 mM KCl, 1.8 mM KH_2_PO_4_, pH 8.0) with 2 equiv of the corresponding PNAs **6**–**11**, **18** and **19** placed in 96-well plates and covered with paraffin oil; fluorescence intensities were measured every 5 min during 12 h at 30 °C; PNA code: **6**: blue, **7**: green, **8**: maroon, **9**: brown, **10**: purple, **11**: coral, **18**: yellow and **19**: gray; solid line for *trans-*isomers and dashed line for *cis-*isomers. Black lines controls: solid: FAM-ssDNA without PNA and under the same conditions; dashed: BHQ/FAM-dsDNA.

Regarding the photoswitchable PNAs, we observed a correlation between the *T*_M_ and the increase of fluorescence in the displacement-strand assays. Thus, the general trend is: the higher the *T*_M_ is, the better is their strand-exchange ability (higher exchange and faster) (Table S3, Supporting Information File 1). PNAs **10** and **11** bearing two and three *o*F_4_Azo moieties, respectively, did not cause an increase of fluorescence. While such result was expected for **11**, according to the melting experiments, the unfortunate outcome of **10** could be probably attributed to its low *T*_M_ in comparison with the quenched dsDNA one (≈40 °C versus 61.5 °C; Table 2, Figure S37, Supporting Information File 1). This would also explain the incomplete displacement (<50%, Table 3, left) for all modified PNAs. In order to improve our method, we tested a shorter 11-mer FAM-ssDNA provided with a toehold under lower temperatures (30 °C). This overhang together with the length of the labelled oligonucleotide must accelerate the strand exchange [53]. Indeed, the displacement was facilitated, reaching almost quantitative exchanged (70–85%; Table 3, right) with faster kinetics (Figure 5B) without compromising the specificity of the system. More importantly, this new set up led to slightly higher differences between isomers and pointed out the dual-labelled probe **10** as the best one, which is consistent with the melting experiments (Table 2 and Figure 3). Among the single mutated PNAs, those with lower *T*_M_, displayed the best performance in our kinetics studies (Figure 5B).

Finally, in an attempt of gaining insight into the general low photoresponsivity, we performed CD (Figure S53, Supporting Information File 1) and UV–vis (Figures S54 and S55, Supporting Information File 1) experiments. The joint-evaluation of these results suggested that the photoswitch is located inside of the PNA–DNA duplex (Figure S53, Supporting Information File 1) but it may not effectively intercalate since pre-hybridization did not affect the isomerization efficiency (Figure S55, Supporting Information File 1). This would explain the low observed photoresponsivity.

## Conclusion

We have successfully synthesized two novel types of visible-light photoresponsive PNAs by coupling on-resin the corresponding molecular transducer. In particular, we focused on the tetra-*ortho*-fluoroazobenzene (*o*F_4_Azo) and the hemithioindigo (HTI) photoswitches; the latter has not been studied in the context of photoregulation of oligonucleotides before. The UV–vis measurements of these probes suggested that the PNA(*o*F_4_Azo) displayed superior photochemical properties to control oligonucleotide hybridization by irradiation. Thus, for the case of the PNA(HTI) just 47% of *trans-*isomer was detected upon irradiation at 405 nm. In addition, the stability of this isomer is compromised. On the contrary, PNA(*o*F_4_Azo) had better conversions and high stability of the non-thermodynamically isomer, even at 90 °C. Both melting experiments and strand displacement assays demonstrated that mismatches had a dramatic effect on the binding affinity of the PNA, which was slightly compensated by the incorporation of *o*F_4_Azo. We observed modest, yet clear, differences in the formation of PNA/DNA duplexes depending on the number and the localization of the *o*F_4_Azo. Further work on increasing the photoresponsivity by exploring different connectors and approaches beyond the base-surrogate approaches is necessary. However, we first demonstrated the great potential *o*F_4_Azo in the context of both PNA and oligonucleotide hybridization. Thus, we believe that the excellent photochemical properties of the *o*F_4_Azo together with the use of the visible-light irradiation may overcome some of the current limitations for in vivo photoregulation of gene expression and related enzymatic reactions in the near future. Besides, having at our disposal such antisense probes, whose activation is reversibly controlled, will contribute to the deciphering of biological pathways. Furthermore, the exceptional stability of the *cis*-isomer may open new venues of this artificial photoswitchable oligonucleotide in other fields different from life science such as nanotechnology and materials science.

## Supporting Information

File 1 Detailed experimental procedures, synthesis, characterization data.
