# Supplementary material for: In search of visible-light photoresponsive peptide nucleic acids (PNAs) for reversible control of DNA hybridization
Source: Beilstein J Org Chem. 2019 Oct 22;15:2500–8. doi: 10.3762/bjoc.15.243 (PMC6839558; doi:10.3762/bjoc.15.243)
Supplement: File 1 — Detailed experimental procedures, synthesis, characterization data. [file Beilstein_J_Org_Chem-15-2500-s001.pdf]

BEILSTEIN JOURNAL OF ORGANIC CHEMISTRY

Supporting Information

for

In search of visible-light photoresponsive peptide nucleic acids (PNAs) for reversible control of DNA hybridization

Lei Zhang, Greta Linden and Olalla Vázquez

Beilstein J. Org. Chem. **2019,** *15,* 2500–2508. doi:10.3762/bjoc.15.243

Detailed experimental procedures, synthesis, characterization data

License and Terms: This is a supporting information file under the terms of the Creative Commons Attribution License (http://creativecommons.org/licenses/by/4.0). Please note that the reuse, redistribution and reproduction in particular requires that the authors and source are credited.
The license is subject to the *Beilstein Journal of Organic Chemistry* terms and conditions: (https://www.beilstein-journals.org/bjoc)

Table of Contents

Abbreviations S1
Experimental procedures.. S3
Synthesis of photoswitches S6
Solid-phase synthesis... S13
Photoisomerization of photoswitchable PNAs.. S22
Studies of PNA/DNA hybridization.. S30

Abbreviations

Å	angstrom
°C	degrees Celsius
A	adenosine
a	Aeg(adenine)
Aeg	*N*-(2-aminoethyl)glycine
Alloc	allyloxycarbonyl
AU	absorbance unit
arom	aromatic
Azo	azobenzene
br	broadened (spectral)
C	cytidine
CD	circular dichroism
c	concentration<br>Aeg(cytosine)
calcd	calculated
δ	NMR chemical shift in parts per million downfield from a standard (tetramethylsilane)
d	doublet (spectral)
DIC	*N,N'*-diisopropylcarbodiimide
DIPEA	*N,N*-diisopropylethylamine
DMSO	dimethyl sulfoxide
DNA	deoxyribonucleic acid
ε	molar extinction coefficient
EI	electron ionization
equiv	equivalent
ESI	electrospray ionization (mass spectrometry)
et al.	and others
Fmoc-OSu	(2,5-dioxopyrrolidin-1-yl) 9*H*-fluoren-9-ylmethyl carbonate
G	guanosine
g	Aeg(guanine)
HPLC	high-performance liquid chromatography
HRMS	high-resolution mass spectrometry
HTI	hemithioindigo
Hz	Hertz
lm	lumen
J	coupling constant (NMR spectrometry)
K	Kelvin
λ	wavelength
LED	light-emitting diode
m	milli (10^{-3})<br>multiplet (spectral)
M	molar (moles per liter)
M^+/M^-	molecular ion
m/*z*	mass-to-charge ratio
MeOH	methanol
MS	mass spectrometry
NMM	4-methylmorpholine
NMR	nuclear magnetic resonance
oF_4Azo	tetra-*ortho*-fluoroazobenzene
PBS	phosphate-buffered saline
PNA	peptide nucleic acid
ppm	parts per million

PyBOP	benzotriazol-1-yl-oxytripyrrolidinophosphonium hexafluorophosphate
q	quaternary
R_f	retention factor
RP	reversed-phase
s	singlet (spectral)
T	5-methyluridine
t	triplet (spectral)<br>Aeg(thymine)
TEA	*N,N*-diethylethanamine
TFA	trifluoroacetic acid
TLC	thin-layer chromatography
UV	ultraviolet
UV–vis	ultraviolet–visible
V	volt
v/v	volume per volume
W	watt
w/v	weight per volume

Experimental procedures

Materials and experimental procedures

All commercial reagents were purchased from the following companies and used without further purification: NaCl biograde and DIPEA from Carl Roth (Germany); Fmoc-OSu and Oxyma from Carbolution (Germany); thionyl chloride, 2-(carboxymethylthio)benzoic acid and 4-methylmorpholine from Alfa Aesar (Germany); DIC from Acros (USA); 2,4-difluoroaniline, Oxone® and PyBOP from Novabiochem (Germany); aniline, 4-bromo-2,6-difluoraniline from Fluorochem (UK); 2,6-lutidine, phenylsilane, copper(I) cyanide and HCl from TCI (Japan); 4-aminobenzoic acid, aluminium trichloride, DMSO biograde, palladium(II) acetate, *tert*-butyl-2-bromoacetate, ethylenediamine, allyl chloroformate and TFA from Sigma Aldrich/Merck (Germany); Fmoc-protected amino acids, piperidine, DMF peptide grade from Iris Biotech (Germany); MeCN HPLC grade, triethylamine and paraffin oil from VWR (France). TentaGel® S RAM resin from RAPP Polymere (Germany); Fmoc-PNA monomers from PNABio (USA); DNA (standard desalting or HPLC purified respectively) from IDT (USA). Water was purified with a Milli-Q Ultra Pure Water Purification System (TKA, Germany).

Nuclear magnetic resonance spectroscopy (NMR)

NMR spectra were recorded at 300 K either on a Bruker AV III HD 300 MHz at a frequency of 300 MHz (^{1}H), 75 MHz (^{13}C) or on a Bruker AV III HD 500 MHz at a frequency of 500 MHz (^{1}H), 125 MHz (^{13}C). The ^{1}H and ^{13}C NMR spectra were referenced to solvent residue peaks. As internal standards, deuterated chloroform ($CDCl_3$) or deuterated dimethyl sulfoxide (DMSO-d_6) with TMS were used. Solvent shifts (ppm): ($CDCl_3$, δ) = 7.26 ppm (^{1}H) and 77.16 ppm (^{13}C), (DMSO-d_6, δ) = 2.50 ppm (^{1}H), and 39.52 ppm (^{13}C).[1]

Mass spectrometry

High resolution electrospray ionization (ESI) mass spectra were acquired with a LTQ-FT Ultra mass spectrometer (Thermo Fischer Scientific). Electron ionization (EI) was performed on an AccuTOF GCv (JEOL).

UV–vis spectroscopy

Concentration determinations and UV-vis measurements were performed on a Tecan (Switzerland) Spark 20M multimode microplate reader at room temperature for the photoswitchable building blocks and yield quantification of PNA compounds by applying the corresponded molar extinction coefficient of the measured compound with Lambert-Beer law:

$$A = \varepsilon \cdot c \cdot l$$

where A = absorbance; ε = molar extinction coefficient; c = concentration; l = path length.

UV–vis measurements at elevated temperature were performed on a Jasco V-650 UV–vis spectrophotometer equipped with a Jasco PAC-743 temperature-controlled cell holder and a Julabo F250 cooling system. All steady-state measurements were performed with 1 mL sample in a 1400 µL quartz cuvette (Hellma Analytics (104F-QS)) with a pathlength of 1 cm.

Melting temperature determination

Melting curves were performed on a Jasco V-650 UV–vis spectrophotometer equipped with a Jasco PAC-743 temperature-controlled cell holder and a Julabo F250 cooling system to maintain the temperature from 20 °C to 95 °C during the measurements. The absorbance was recorded at 260 nm.

High-performance liquid chromatography

For preparative purposes, Varian (USA) ProStar Preparative HPLC System with a preparative Juptier 10 u C18 300 Å column (10 µm, 250 × 10 mm; Phenomenex) with a flow rate of 8 mL/min was used. An UV detector at wavelength of 220 nm and 260 nm was used for signal detection. The eluents were Milli-Q water (A) and MeCN (B) with addition of 0.1% TFA.

For analytical purposes, an Agilent 1260 Infinity II HPLC-System (Agilent Technologies, USA) with an eclipse XDB-C18 column (5 µm, 4.6 × 150 mm, Agilent) with a flow rate of 1.0 mL/min was used. An UV detector at wavelength of 220 nm and 260 nm was used for signal detection. The eluents were Milli-Q water with addition of 0.05% TFA (A) and MeCN with the addition of 0.03% TFA (B).

For isomer ratio quantification, a kinetex column (5 µm XB-C18 100 Å, 150 × 4.6 mm, Phenomenex) with a flow rate of 1.0 mL/min was used. An UV detector at isosbestic wavelength of 275 nm for *ortho*-fluoroazobenzene and hemithioindigo derivative was used for signal detection. The eluents were Milli-Q water with addition of 0.05% TFA (A) and MeCN with the addition of 0.03% TFA (B).

Photoisomerization

An UV-emitter 405 nm SMD (Star-UV405-03-00-00, 3 W, Roschwege, Germany) and a HighPower-LED Green (87 lm, 130°, 3.8 V, 1000 mA, Roschwege) were used for irradiation at 405 nm and 525 nm, respectively. For the irradiation at 525 nm an additional cut-on filter (OG515 colored glass filter, 515 nm longpass, Thorlabs, USA) was applied. Benda (type: NU-4 KL) lamp and a Luminea spot LED E27 lamp were used for irradiation at 365 and 430 nm.

Fluorescence measurements

Quenching effect study of PNA on FAM was performed in 0.2 mL non-skirted 96-well PCR plates sealed with adhensive sealing sheets (Thermo Fischer Scientific, USA) in a Mx3000P QPCR system (Agilent Technologies, USA).

Strand-displacement experiments were assessed in black clear bottom 96 well plates (µclear, Greiner Bio-one, Austria) by recording fluorescence emission at 520 nm in a Spark 20M platereader (Tecan, Switzerland) at 30 °C as well as 37 °C and 460 nm excitation wavelength. The measurements were performed with the following settings: integration time: 40 µs; excitation bandwidth: 15 nm; emission bandwidth: 25 nm.

Circular Dichroism

CD measurements were performed on a Jasco J-810 Spectropolarimeter equipped with a Jasco CDF-426S peltier controller and a Thermo Haake WKL 26 water recirculator at 20 °C using a CD cuvette (Hellma Analytics; QS 0.100). The settings were: continuous scanning mode, range from 360–220 nm, speed of 100 nm/min, response of 0.25 s, band width of 2 nm, data pitch of 0.2 nm and 5-10 accumulations per sample with standard sensitivity. The represented measurements were always buffer subtracted.

Synthesis of photoswitches

Synthesis of *ortho*-fluoroazobenzene-4-carboxylic acid

The synthesis was mainly followed the procedures of Appiah et al. [2] and Knie et al .[3]

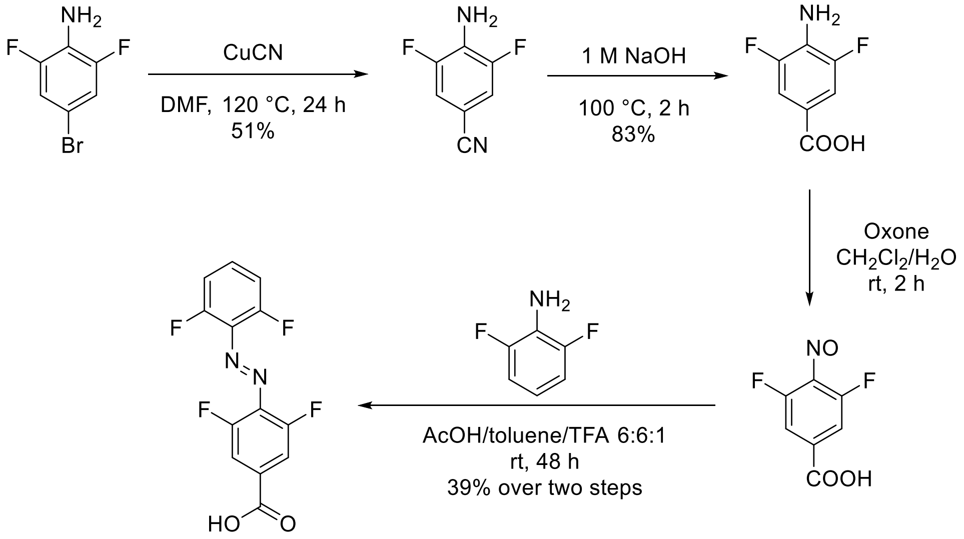

Figure S1: Synthesis route of photoswitch ***ortho*-fluoroazobenzene-4-carboxylic acid** (***o*F$_4$Azo**).

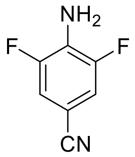

4-Amino-3,5-difluorobenzonitrile: 4-Bromo-2,6-difluoroaniline (5.00 g, 24.0 mmol, 1.00 equiv) and copper(I) cyanide (6.45 g, 72.0 mmol, 3.00 equiv) were suspended in 50 mL DMF and heated at 120 °C for 24 h. After cooling to room temperature, the mixture was poured into an aqueous solution of ammonia (12%, v%, 250 mL). The mixture was then filtered and the precipitate was washed with EtOAc (500 mL). The organic phases were combined and washed with the ammonia solution (500 mL), distilled water (500 mL), brine (500 mL) and dried over anhydrous $MgSO_4$. Solvents were removed under reduced pressure. The crude was purified by flash column chromatography (CH_2Cl_2/*n*-pentane, 2:1, v/v) to yield the desired compound (1.88 g, 12.2 mmol, 51%) as a white solid. **TLC:** R_f = 0.39 (CH_2Cl_2/*n*-pentane, 2:1, v/v). The characterization is in agreement with the literature.[2] **^{1}H-NMR (300 MHz, $CDCl_3$, *δ*):** 7.13 (dd, ^3J(H,F) = 6.0 Hz, ^4J(H,H) = 2.2 Hz, 2H; 2×H_{arom}), 4.29 (s, 2H; NH_2) ppm. **^{13}C-NMR (75 MHz, $CDCl_3$, *δ*):** 152.3 (C_{arom}), 149.1 (C_{arom}), 129.7(C_{arom}), 118.0 (*C*N), 115.7 (C_{arom}H), 115.4 (C_{arom}H); 98.4 (C_{arom}) ppm. **HRMS-EI (m/z):** $[M]^+$ calcd for $C_7H_4F_2N_2$, 154.03425; found, 154.03252.

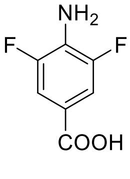

4-Amino-3,5-difluorobenzoic acid: 4-Amino-3,5-difluorobenzonitrile (1.00 g, 6.49 mmol, 1.00 equiv) was suspended in 1 M NaOH aqueous solution (80 mL) and heated to reflux for 4 h. The reaction mixture was cooled to room temperature and acidified with 1 M HCl aqueous solution (50 mL). The resulting precipitate was filtered, washed with distilled water (200 mL) and dried to yield the desired product (0.93 g,

5.39 mmol, 83%) as a white solid. The characterization is in agreement with the literature.[2] **TLC:** R_f = 0.10 (EtOAc). **^{1}H-NMR (300 MHz, DMSO-*d*$_6$, *δ*):** 12.68 (s, 1H; COO*H*), 7.40 (dd, ^3J(H,F) = 4.2 Hz, ^4J(H,H) = 1.4 Hz, 2H; 2×H_{arom}), 6.05 (s, 2H; NH_2) ppm. **^{13}C-NMR (75 MHz, DMSO-*d*$_6$, *δ*):** 166.0 (*C*OOH), 151.3 (C_{arom}), 148.2 (C_{arom}), 130.6 (C_{arom}), 115.6 (C_{arom}), 112.3 (C_{arom}H), 112.1 (C_{arom}H) ppm. **HRMS-ESI (m/z):** [M-H]$^-$ calcd for $C_7H_4F_2NO_2$, 172.0216; found, 172.0217.

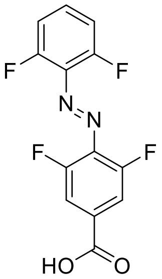

***ortho*-Fluoroazobenzene-4-carboxylic acid**: 4-Amino-3,5-difluorobenzoic acid (500 mg, 2.89 mmol, 1.00 equiv) was dissolved in CH_2Cl_2/acetone (20 mL, 5:1, v/v), a solution of Oxone® (3.56 g, 5.78 mmol, 2.00 equiv) in distilled water (10.0 mL) was added and the resulting mixture was stirred at room temperature for 4 h. The organic layer was washed with distilled water (100 mL), and brine (100 mL), dried over anhydrous $MgSO_4$. Solvents were removed under reduced pressure. The residue was suspended in a mixture of acetic acid/toluene/TFA (40 mL, 6:6:1, v/v/v). 2,6-Difluoroaniline (299 mg, 2.31 mmol, 0.80 eq) was added and the resulting mixture was stirred at room temperature for 2 days. The reaction was diluted with distilled water (200 mL) and extracted with EtOAc (3 × 200 mL). The organic layer was dried over anhydrous $MgSO_4$ and solvents removed under reduced pressure. The crude product was purified by flash column chromatography (*n*-pentane/EtOAc, 1:3, v/v) to yield the desired compound (336 mg, 1.13 mmol, 39%) as orange solid. **TLC:** R_f = 0.50 (*n*-pentane/EtOAc, 1:3, v/v). The characterization is in agreement with the literature.[3] **^{1}H-NMR (300 MHz, DMSO-*d*$_6$, *δ*):** 13.86 (s, 1H; COO*H*), 7.80 (d, ^3J(H,F) = 9.2 Hz, 2H; 2×H_{arom}), 7.74-7.64 (m, 1H; 1×H_{arom}), 7.43-7.38 (m, 2H; 2× H_{arom}) ppm. **^{13}C-NMR (75 MHz, DMSO-*d*$_6$, *δ*):** 164.6 (*C*OOH), 155.9 (C_{arom}F), 155.2 (C_{arom}F), 153.8 (C_{arom}F), 153.1 (C_{arom}F), 134.5 (C_{arom}), 134.2 (C_{arom}), 133.3 (C_{arom}), 130.5 (C_{arom}), 114.0 (C_{arom}H), 113.8 (C_{arom}H), 113.5 (C_{arom}H), 113.3 (C_{arom}H) ppm. **HRMS-ESI (m/z):** [M-H]$^-$ calcd for $C_{13}H_5F_4N_2O_2$, 297.0282; found, 297.0291.

Synthesis of 4-(phenylazo)benzoic acid

The synthesis was followed the procedures of Knie et al. [3] and Meng et al. [4]

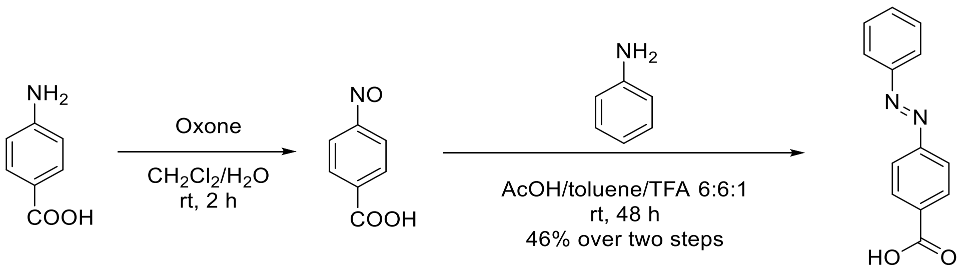

Figure S2: Synthesis route of photoswitch **4-(phenylazo)benzoic acid** (**Azo**).

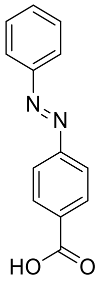

4-(Phenylazo)benzoic acid: 4-Aminobenzoic acid (1.00 g, 7.29 mmol, 1.00 equiv) was dissolved in CH_2Cl_2/acetone (50 mL, 5:1, v/v), a solution of Oxone® (2.22 g, 14.6 mmol, 2.00 equiv) in water (20.0 mL) was added and the resulting mixture was stirred at room temperature for 2 h. The organic layer was washed with distilled water (100 mL), and brine (100 mL), dried over anhydrous $MgSO_4$. Solvents were removed under reduced pressure. The residue was suspended in a mixture of acetic acid/toluene/TFA (50 mL, 6:6:1, v/v/v). Aniline (0.53 mL, 5.83 mmol, 0.80 equiv) was added and the resulting mixture was stirred at room temperature for 24 h. The reaction was diluted with distilled water (200 mL) and extracted with EtOAc (3 × 200 mL). The organic layer was dried over anhydrous $MgSO_4$ and solvents removed under reduced pressure. The crude product was purified by flash column chromatography (*n*-pentane/EtOAc, 1:2, v/v) to yield the desried compound (750 mg, 3.32 mmol, 46%) as orange solid. The characterization is in agreement with the literature.[4] **TLC:** R_f = 0.32 (*n*-pentane/EtOAc, 1:2, v/v). **^{1}H-NMR (300 MHz, DMSO-d_6, δ):** 13.24 (s, 1H; COO*H*), 8.17-7.13 (m, 2H; 2×H_{arom}), 7.98-7.92 (m, 4H; 4×H_{arom}), 7.65-7.60 (m, 3H; 3×H_{arom}) ppm. **^{13}C-NMR (75 MHz, DMSO-d_6, δ):** 166.6 (*C*OOH), 154.2 (C_{arom}), 151.9 (C_{arom}), 132.8 (C_{arom}), 132.1 (C_{arom}H), 130.6 (2C; 2×C_{arom}H), 129.5 (2C; 2×C_{arom}H), 122.8 (2C; 2×C_{arom}H), 122.5 (2C; 2×C_{arom}H) ppm. **HRMS-ESI (m/z):** $[M-H]^-$ calcd for $C_{13}H_9N_2O_2$, 225.0670; found, 225.0672.

Synthesis of 3-oxo-2-[1'-phenylmethylidene]-2,3-dihydrobenzo[*b*]thiophene-7-carboxylic acid

The synthesis was followed the procedure of Varedian et al. [5]

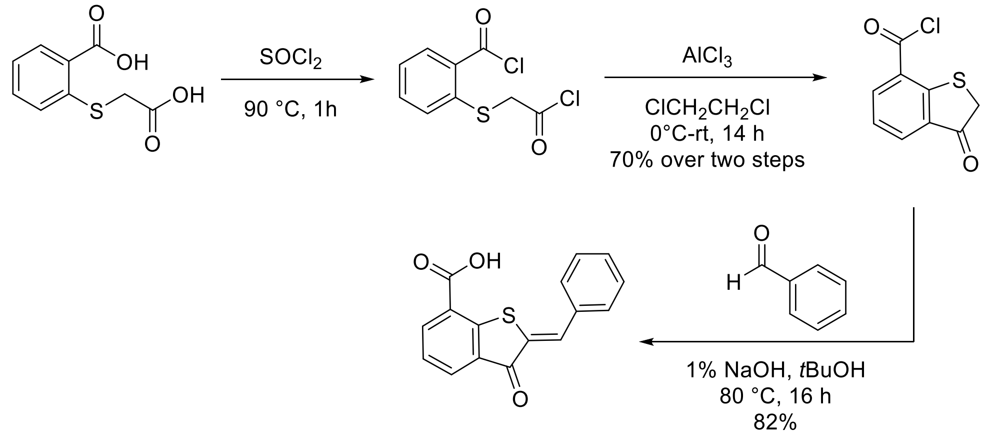

Figure S3: Synthesis route of photoswitch ***hemithioindigo*** (**HTI**).

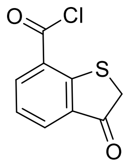

3-Oxo-2,3-dihydrobenz[*b*]thiophene-7-carbonylchloride: (*o*-Carboxyphenylthio)acetic acid (300 mg, 1.41 mmol, 1.00 equiv) was dissolved in $SOCl_2$ (30 mL) and heated to reflux for 1 h. The reaction mixture was cooled to room temperature and the excess of $SOCl_2$ was distilled under reduced pressure. The residue was dissolved in 1,2-dichloroethane (30 mL) under nitrogen atmosphere and cooled to 0 °C. $AlCl_3$ (471 mg, 3.53 mmol, 2.50 equiv) was added in several portions and the mixture was stirred for another 30 min at 0 °C. The reaction was then allowed to warm to room temperature and stirred for another 14 h. The reaction mixture was quenched with ice water (100 mL) and extracted with CH_2Cl_2 (3 × 100 mL). The combined organic layer was dried over anhydrous Na_2SO_4 and concentrated under reduced pressure. The residue was dried in vacuum to yield the desired compound (214 mg, 0.99 mmol, 70%) as yellow solid. The characterization is in agreement with the literature.[5] **^{1}H-NMR (300 MHz, $CDCl_3$, *δ*):** 8.52 (dd, ^3J(H,H) = 7.8 Hz, ^4J(H,H) = 1.3 Hz, 1H; H_{arom}), 8.05 (dd, ^3J(H,H) = 7.6 Hz, ^4J(H,H) = 1.3 Hz, 1H; H_{arom}), 7.42 (t, ^3J(H,H) = 7.7 Hz, 1H; H_{arom}), 3.82 (s, 2H; CH_2) ppm. **^{13}C-NMR (75 MHz, $CDCl_3$, *δ*):** 198.8 (*C*O), 166.2 (*C*OCl), 158.3 (C_{arom}), 141.0 (C_{arom}), 133.3 (C_{arom}H), 132.7 (C_{arom}H), 128.7 (C_{arom}), 125.3 (C_{arom}H), 39.9 (CH_2) ppm. **HRMS-EI (m/z):** $[M]^+$ calcd for $C_9H_5ClO_2S$, 211.96988; found, 211.97009.

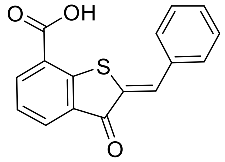

3-Oxo-2-[1'-phenylmethylidene]-2,3-dihydrobenzo[*b*]thiophene-7-carboxylic acid: 3-Oxo-2,3-dihydrobenz[*b*]thiophene-7-carbonyl chloride (200 mg, 0.94 mmol, 2.10 equiv) was dissolved in a mixture of 1% NaOH aqueous solution (5 mL, w/v) and *t*-BuOH (10 mL) under nitrogen atmosphere. The mixture was cooled to 0 °C and benzaldehyde (46 µL, 0.45 mmol, 1.00 equiv)

was added. The resulting mixture was stirred at 0 °C for 40 min and then refluxed for another 16 h. After cooling to room temperature, the reaction mixture was acidified with acetic acid until pH 2 was reached. The reaction was diluted with distilled water (100 mL) and extracted with EtOAc (3 × 100 mL). The organic layer was dried over anhydrous Na_2SO_4 and concentrated under reduced pressure The crude product was purified by column chromatography (*n*-pentane/EtOAc, 1:1, v/v) to yield the desired compound (104 mg, 0.37 mmol, 82%) as yellow powder. The characterization is in agreement with the literature.[5] **TLC:** R_f = 0.50 (*n*-pentane/EtOAc, 1:3, v/v). **^{1}H-NMR (300 MHz, DMSO-d_6, δ):** 8.27 (dd, ^3J(H,H) = 7.5 Hz, ^4J(H,H) = 1.4 Hz, 1H; H_{arom}), 8.00 (dd, ^3J(H,H) = 7.0 Hz, ^4J(H,H) = 1.4 Hz, 1H; H_{arom}), 7.89 (s, 1H; C=C*H*), 7.87 (s, 1H; H_{arom}), 7.84 (s, 1H; H_{arom}), 7.64-7.43 (m, 4H; 4×H_{arom}) ppm. **^{13}C-NMR (75 MHz, DMSO-d_6, δ):** 188.7 (*C*O), 167.4 (*C*OOH), 146.5 (C_{arom}H), 136.4 (C_{arom}), 134.3 (C_{arom}H), 133.5 (C_{arom}), 132.9(C_{arom}H), 132.1 (C_{arom}H), 130.9 (C_{arom}H), 130.6 (C_{arom}H), 130.1 (C_{arom}H), 129.2 (2C, 2×C_{arom}H), 127.4 (2C, 2×C_{arom}H), 125.5 (C=*C*H) ppm. **HRMS-ESI (m/z):** $[M+Na]^+$ calcd for $C_{16}H_{10}O_3SNa$, 305.0243; found, 305.0244.

Synthesis of *tert*-butyl *N*-[(allyloxy)carbonyl]-*N*-(2-{[(9*H*-fluoren-9-ylmethoxy) carbonyl]amino}ethyl) glycinate

The synthesis was followed the procedure of *Bondebjerg et. al.*[6]

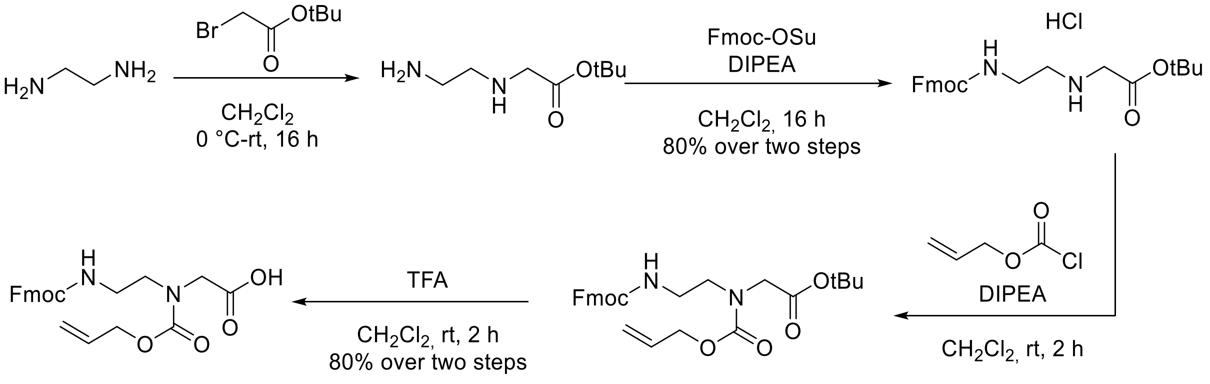

Figure S4: Synthesis route of **Fmoc-Aeg(Alloc)-COOH**.

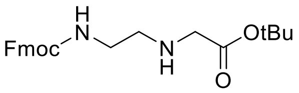

Fmoc-Aeg-*t*-Bu·HCl: Ethylenediamine (10.1 mL, 452 mmol, 20.0 equiv) was dissolved in CH_2Cl_2 (500 mL) and cooled to 0 °C. A solution of *tert*-butyl bromoacetate (1.12 mL, 8.60 mmol, 1.00 equiv) in CH_2Cl_2 (200 mL) was added dropwise over 2 h. The resulting mixture was allowed to warm to room temperature and stirred for another 16 h. The reaction mixture was washed with water (3 × 500 mL). The organic layer was dried over anhydrous Na_2SO_4 and concentrated under reduced pressure. The crude product was obtained as yellowish oil and was used without further purification. The crude *tert*-butyl-(2-aminoethyl)glycinate (1.50 g, 8.60 mmol, 1.00 equiv) was dissolved in CH_2Cl_2 (500 mL). A solution of Fmoc-OSu (2.76 g, 8.18 mmol, 0.95 equiv) in CH_2Cl_2 (200 mL) was added dropwise and the resulting mixture was stirred for 16 h at room temperature. The reaction mixture was washed with 1 M aqueous HCl solution (3 × 500 mL) and brine (500 mL). The organic layer was dried over anhydrous Na_2SO_4 and concentrated under reduced pressure. The residue was suspended in acetone (50 mL) and added into *n*-pentane (200 mL). The white precipitate was collected and washed with *n*-pentane (500 mL). The desired compound (3.16 g, 7.31 mmol, 85%) was obtained as white solid. The characterization is in agreement with the literature.[6] **^{1}H-NMR (300 MHz, DMSO-*d*$_6$, *δ*):** 9.33 (br, 1H; N*H*), 7.89 (d, ^3J(H,H) = 7.4 Hz, 2H; 2×H_{arom}), 7.70 (d, ^3J(H,H) = 7.4 Hz, 2H; 2×H_{arom}), 7.57 (t, ^3J(H,H) = 5.5 Hz, 1H; N*H*), 7.42 (t, ^3J(H,H) = 7.1 Hz, 2H; 2×H_{arom}), 7.33 (t, ^3J(H,H) = 6.9 Hz, 2H; 2×H_{arom}), 4.33 (d, ^3J(H,H) = 6.5 Hz, 2H; CH_2), 4.23 (t, ^3J(H,H) = 6.7 Hz, 1H; C*H*), 3.86 (s, 2H; CH_2), 3.41-3.27 (m, 2H, CH_2), 3.00 (t, ^3J(H,H) = 6.3 Hz, 2H; CH_2), 1.46 (s, 9H; 3×CH_3) ppm. **^{13}C-NMR (75 MHz, DMSO-*d*$_6$, *δ*):** 165.6 (*C*OO), 156.2 (HN*C*OO), 143.8 (2C; 2×C_{arom}), 140.7 (2C; 2×C_{arom}), 127.6 (2C; 2×C_{arom}H), 127.0 (2C; 2×C_{arom}H), 125.1 (2C; 2×C_{arom}H), 120.1 (2C; 2×C_{arom}H), 83.0 (C_q), 65.6 (O*C*H$_2$), 47.2 (*C*H$_2$), 46.6 (*C*H$_2$), 36.6 (*C*H), 30.6 (*C*H$_2$), 27.6 (3C; 3×*C*H$_3$) ppm. **HRMS-ESI (m/z):** $[M+H]^+$ calcd for $C_{23}H_{29}N_2O_4$, 398.2154; found, 398.2161.

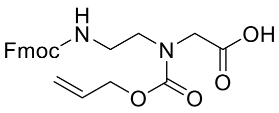

Fmoc-Aeg(Alloc)-COOH: Fmoc-Aeg-*t*-Bu·HCl (1.50 g, 3.46 mmol, 1.00 equiv) and allyl chloroformate (737 µL, 6.93 mmol, 2.00 equiv) were dissolved in CH_2Cl_2 (500 mL). A solution of DIPEA (1.21 mL, 6.93 mmol, 2.00 equiv) in CH_2Cl_2 (50 mL) was added dropwise and the resulting solution was stirred at room temperature for 2 h. The reaction mixture was washed with 0.1 M aqueous HCl solution (500 mL) and brine (500 mL). The organic layer was dried over anhydrous $MgSO_4$ and concentrated under reduced pressure. The residue was dissolved in a mixture of CH_2Cl_2 (10 mL) and TFA (10 mL) and stirred at room temperature for 1 h. The solvents were coevaporated with toluene (3 × 100 mL). The residue was dissolved in EtOAc (10 mL) and precipitated in *n*-hexane (100 mL). The precipitate was collected and washed with *n*-hexane resulting in the desired compound (1.17 g, 2.77 mmol, 80%) as white solid. The characterization is in agreement with the literature.[6] **^{1}H-NMR (300 MHz, DMSO-*d*$_6$, *δ*):** 12.72 (s, 1H; COO*H*), 7.89 (d, ^3J(H,H) = 7.4 Hz, 2H; 2×H_{arom}), 7.67 (d, ^3J(H,H) = 7.4 Hz, 2H; 2×H_{arom}), 7.41 (t, ^3J(H,H) = 7.1 Hz, 2H; 2×H_{arom}), 7.37-7.32 (m, 3H; 2×H_{arom}, N*H*), 6.05-5.70 (m, 1H; C*H*=CH$_2$), 5.37-5.05 (m, 2H; CH=CH_2), 4.54-4.44 (m, 2H; CH_2), 4.37-4.13 (m, 4H; 2×CH_2), 3.91 (d, ^3J(H,H) = 9.5 Hz, 1H; C*H*), 3.35-3.25 (m, 2H; CH_2), 3.21-3.08 (m, 2H; CH_2) ppm. **^{13}C-NMR (75 MHz, DMSO-*d*$_6$, *δ*):** 171.3 (COOH), 156.1 (NH*C*OO), 155.5 (N*C*OO), 143.9 (2C; 2×C_{arom}), 140.7 (2C; 2×C_{arom}), 133.2 (*C*H=CH$_2$), 127.6 (2C; 2×C_{arom}H), 127.1 (2C; 2×C_{arom}H), 125.2 (2C; 2×C_{arom}H), 125.1, 120.2 (2C; 2×C_{arom}H), 116.7 (CH=CH$_2$), 66.4 (CH$_2$), 65.2 (CH$_2$), 49.0 (CH$_2$), 48.8 (CH$_2$), 47.7 (CH$_2$), 47.0 (*C*H) ppm. **HRMS-ESI (m/z):** $[M+Na]^+$ calcd for $C_{23}H_{24}N_2O_6Na$, 447.1527; found, 447.1531.

Solid-phase synthesis

The peptide nucleic acids (PNAs) were synthesized manually according the procedures of Fmoc-based solid-phase peptide synthesis.

General protocol

The synthesis was performed in a 2 mL polypropylene reactor with plunger and frit (25 µm poresize, Multi Syn Tech GmbH). TentaGel® S RAM resin (0.19 mmol/g, RAPP Polymere, Germany) was used as solid support. The amounts of reagents of the following synthesis protocol correspond to 1–5 µmol scale.

Swelling: The resin was swollen in 2 mL DMF for 30 min.

Deprotection of the temporal Fmoc group: The resin was treated twice with 1 mL piperidine (20% in DMF, v%) for 5 min each and subsequently washed with DMF (5 × 2 mL), CH_2Cl_2 (5 × 2 mL) and DMF (5 × 2 mL).

Coupling of Fmoc-PNA monomer: The Fmoc-PNA monomers (4.00 equiv), PyBOP (4.00 equiv) and NMM (8.00 equiv) were dissolved in DMF (0.20 M) and pre-activated for 3 min. The mixture was added to the resin. After 45 min, the resin was washed with DMF (5 × 2 mL), CH_2Cl_2 (5 × 2 mL) and DMF (5 × 2 mL).

Coupling of Fmoc-Aeg(Alloc)-COOH: The Fmoc-Aeg(Alloc)-COOH (4.00 equiv), PyBOP (4.00 equiv) and NMM (8.00 equiv) were dissolved in DMF (0.20 M) and pre-activated for 3 min. The mixture was added to the resin. After 45 min, the resin was washed with DMF (5 × 2 mL), CH_2Cl_2 (5 × 2 mL) and DMF (5 × 2 mL).

Capping: The resin was treated with 1 mL of a solution of 2,6-lutidine/Ac_2O/DMF (6:5:89, v/v/v) for 5 min. The resin was washed with DMF (5 × 2 mL), CH_2Cl_2 (5 × 2 mL) and DMF (5 × 2 mL). After the last coupling, the resin was washed with (5 × 2 mL), CH_2Cl_2 (5 × 2 mL) and finally dried under reduced pressure.

Alloc-deprotection: The resin was swollen in 2 mL DMF for 30 min and washed with CH_2Cl_2 (5 × 2 mL) and purge with nitrogen. $Pd(OAc)_2$ (0.05 equiv) and triphenylphosphine (1.50 equiv) were added to a solution of phenylsilane (20.0 equiv) and NMM (20.0 equiv) in CH_2Cl_2 (0.02 M). Afterwards the resin was washed with sodium diethyldithiocarbamate (5 × 2 mL, 0.5% in DMF, v/v), CH_2Cl_2 (5 × 2 mL) and DMF (5 × 2 mL).

Coupling of photoswitches: After the Alloc-deprotection, the corresponding photoswitch carboxylic acid (4.00 equiv), Oxyma (4.00 equiv) and DIC (4.00 equiv) were dissolved in NMP (0.20 M) and pre-activated for 3 min. The mixture was added to the resin. After 2 h, the resin was washed with DMF (5 × 2 mL), CH_2Cl_2 (5 × 2 mL) and DMF (5 × 2 mL).

Cleavage: The dried resin was treated with a solution of TFA/*m*-cresol/H_2O (90:5:5, v/v/v, 0.5 mL for 1 µmol scale and proportionally for bigger scales). After 2 h, the resin was washed with TFA and the combined eluates were added to dry ice-cold Et_2O (1 mL of Et_2O for each 100 µL of cleavage mixture). Centrifugation at 4 °C yield a precipitate which was with cold Et_2O. The residue was dried under reduced pressure.

Purification: The crude product was dissolved in Milli-Q water or Milli-Q water/MeCN (10:1, v/v) depending on the different solubility. The purification was performed as stated in the experimental procedures (High-performance liquid chromatography, for preparative purposes)

Characterization: The freeze-dried products were identified via analytical RP-HPLC-MS on an Agilent 1260 Infinity II LC system (Agilent Technologies). The eluents were Milli-Q water with addition of 0.05% TFA (A) and MeCN (B) with addition of 0.03% TFA) with a flow rate of 1.0 mL/min.

Gradient I: 5% B for 5 min, 5→40% B in 30 min.
Gradient II: 5% B for 5 min, 5→75% B in 30 min.
Gradient III: 5% B for 5 min, 5→95% B in 30 min.
Gradient IV: 10% B for 5 min, 10→25% B in 30 min.

Ac-Aeg(oF$_4$Azo)-CONH$_2$ (1)

Product (13.6 µmol, 49%) was obtained as a yellow solid. t_R = 17.26 min for *cis*-isomer and 17.75 min for *trans*-isomer (Gradient III). HRMS-ESI (m/z): $[M-H]^-$ calcd for $C_{19}H_{16}F_4N_5O_3$, 438.1195; found, 438.1211.

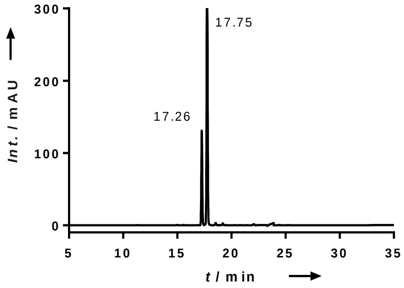

Figure S5: RP-HPLC chromatogram of purified compound **1**, detected at 220 nm.

Ac-Aeg(HTI)-CONH$_2$ (2)

Product (2.60 µmol, 26%) was obtained as a yellow solid. t_R = 22.68 min (Gradient III). HRMS-ESI (m/z): $[M+Na]^+$ calcd for $C_{22}H_{21}N_3O_4S_1Na_1$, 446.1145; found, 446.1148.

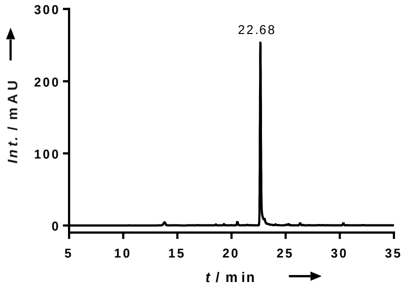

Figure S6: RP-HPLC chromatogram of purified compound **2**, detected at 220 nm.

Ac-ggcag-Aeg(oF$_4$Azo)-gtttct-CONH$_2$ (3)

Product (120 nmol, 12%) was obtained as a yellow solid. t_R = 14.75 min (Gradient II). HRMS-ESI (m/z): $[M+4H]^{4+}$ calcd for $C_{138}H_{168}N_{66}F_4O_{39}$, 862.5783; found, 862.5796.

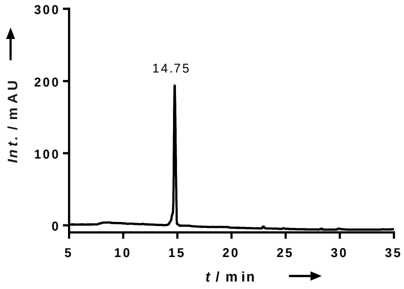

Figure S7: RP-HPLC chromatogram of purified compound **3**, detected at 260 nm.

Ac-ggcag-Aeg(HTI)-gtttct-CONH$_2$ (4)

Product (90.0 nmol, 9%) was obtained as a yellow solid. t_R = 13.95 min (Gradient II). HRMS-ESI (m/z): $[M+5H]^{5+}$ calcd for $C_{141}H_{173}N_{64}O_{40}S_1$, 687.0638; found, 687.0646.

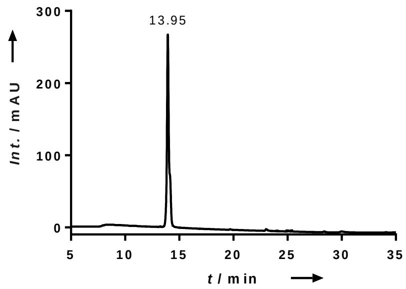

Figure S8: RP-HPLC chromatogram of purified compound **4**, detected at 260 nm.

Ac-ggcag-Aeg(Ac)-gtttct-CONH$_2$ (5)

Product (374 nmol, 15%) was obtained as a white solid. t_R = 11.26 min (Gradient III). HRMS-ESI (m/z): $[M+4H]^{4+}$ calcd for $C_{127}H_{166}N_{64}O_{39}$, 803.0744; found, 803.0775.

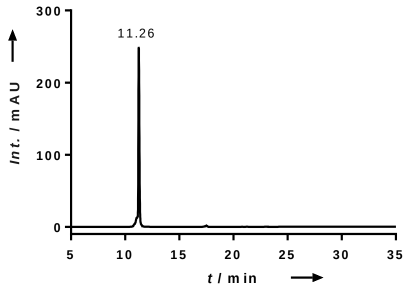

Figure S9: RP-HPLC chromatogram of purified compound **5**, detected at 260 nm.

Ac-K-Aeg(*o*F$_4$Azo)-gcagtgtttcttgg-K-CONH$_2$ (6)

Product (0.27 µmol, 11%) was obtained as a yellow solid. t_R = 14.09 min (Gradient II). HRMS-ESI (m/z): $[M+6H]^{6+}$ calcd for $C_{183}H_{235}N_{8}5O_{52}F_4$, 755.4719; found, 755.4730.

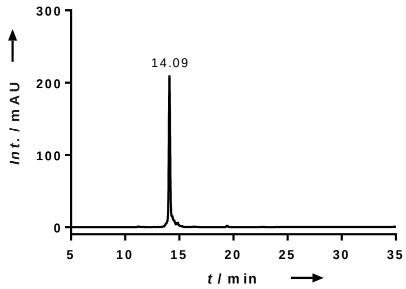

Figure S10: RP-HPLC chromatogram of purified compound **6**, detected at 260 nm.

Ac-K-gg-Aeg(*o*F$_4$Azo)-agtgtttcttgg-K-CONH$_2$ (7)

Product (0.30 µmol, 12%) was obtained as a yellow solid. t_R = 13.88 min (Gradient II). HRMS-ESI (m/z): $[M+6H]^{6+}$ calcd for $C1_{84}H_{235}N_{87}O_{52}F_4$, 762.1395; found, 762.1407.

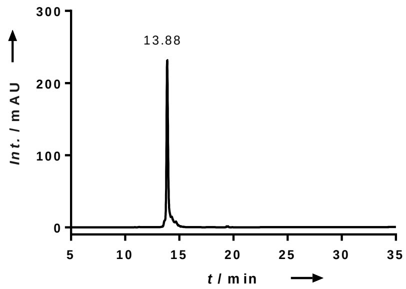

Figure S11: RP-HPLC chromatogram of purified compound **7**, detected at 260 nm.

Ac-K-ggcagt-Aeg(*o*F$_4$Azo)-tttcttgg-K-CONH$_2$ (8)

Product (0.22 µmol, 9%) was obtained as a yellow solid. t_R = 14.54 min (Gradient II). HRMS-ESI (m/z): $[M+6H]^{6+}$ calcd for $C_{183}H_{235}N_{85}O_{52}F_4$, 755.4719; found, 755.4729.

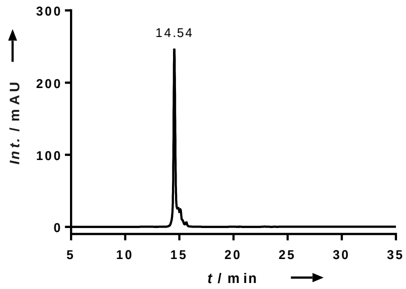

Figure S12: RP-HPLC chromatogram of purified compound **8**, detected at 260 nm.

Ac-K-ggcagtgtttcttg-Aeg(*o*F$_4$Azo)-K-CONH$_2$ (9)

Product (0.20 µmol, 8%) was obtained as a yellow solid. t_R = 14.25 min (Gradient II). HRMS-ESI (m/z): $[M+6H]^{6+}$ calcd for $C_{183}H_{235}N_{8}5O_{52}F_4$, 755.4719; found, 755.4730.

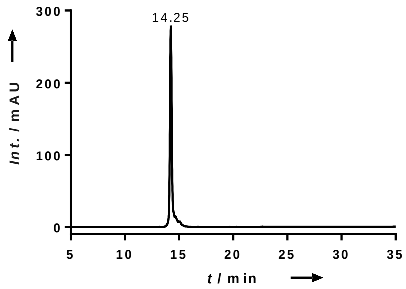

Figure S13: RP-HPLC chromatogram of purified compound **9**, detected at 260 nm.

Ac-K-ggc-Aeg(*o*F_4Azo)-gtgtttc-Aeg(*o*F_4Azo)-tgg-K-$CONH_2$ (10)

Product (0.13 µmol, 5%) was obtained as a yellow solid. t_R = 16.12 min (Gradient II). HRMS-ESI (m/z): $[M+6H]^{6+}$ calcd for $C_{189}H_{233}N_{85}O_{51}F_8$, 777.1357; found, 777.1373.

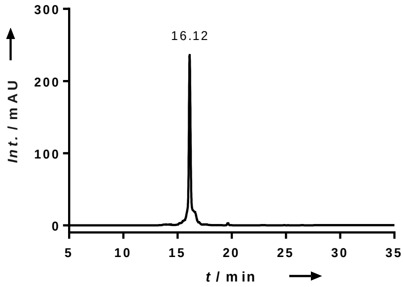

Figure S14: RP-HPLC chromatogram of purified compound **10**, detected at 260 nm.

Ac-K-gg-Aeg(*o*F_4Azo)-agtg-Aeg(*o*F_4Azo)-ttct-Aeg(*o*F_4Azo)-gg-K-$CONH_2$ (11)

Product (0.09 µmol, 4%) was obtained as a yellow solid. t_R = 18.02 min (Gradient II). HRMS-ESI (m/z): $[M+6H]^{6+}$ calcd for $C_{196}H_{231}N_{87}O_{48}F_{12}$, 800.1356; found, 800.1367.

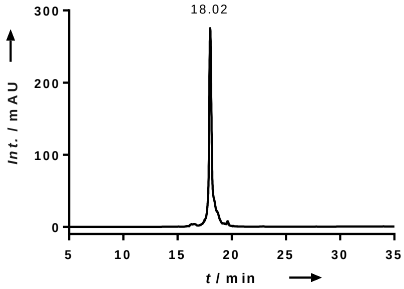

Figure S15: RP-HPLC chromatogram of purified compound **11,** detected at 260 nm.

Ac-K-Aeg(Azo)-gcagtgtttcttgg-K-$CONH_2$ (12)

Product (0.25 µmol, 10%) was obtained as a yellow solid. t_R = 13.47 min (Gradient II). HRMS-ESI (m/z): $[M+6H]^{6+}$ calcd for $C_{183}H_{239}N_{85}O_{52}$, 743.4781; found, 743.4792.

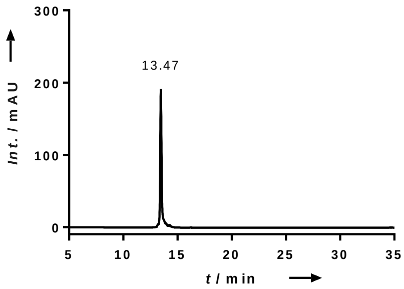

Figure S16: RP-HPLC chromatogram of purified compound **12**, detected at 260 nm.

Ac-K-gg-Aeg(Azo)-agtgtttcttgg-K-CONH$_2$ (13)

Product (0.27 µmol, 11%) was obtained as a yellow solid. t_R = 13.22 min (Gradient II). HRMS-ESI (m/z): $[M+5H]^{5+}$ calcd for $C_{184}H_{239}N_{87}O_{52}$, 900.1751; found, 900.1758.

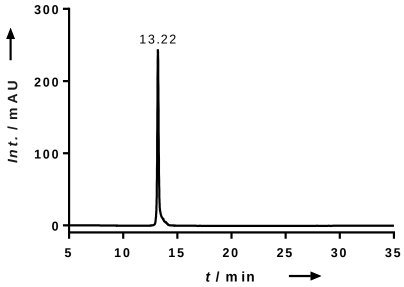

Figure S17: RP-HPLC chromatogram of purified compound **13**, detected at 260 nm.

Ac-K-ggcagt-Aeg(Azo)-tttcttgg-K-CONH$_2$ (14)

Product (0.25 µmol, 10%) was obtained as a yellow solid. t_R = 13.98 min (Gradient II). HRMS-ESI (m/z): $[M+5H]^{5+}$ calcd for $C_{183}H_{238}N_{85}O_{52}$, 891.9723; found, 891.9740.

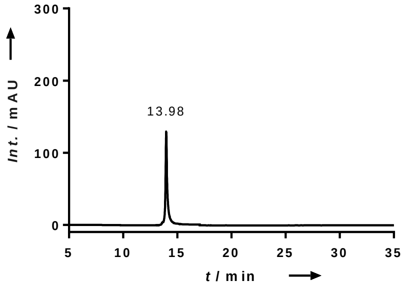

Figure S18: RP-HPLC chromatogram of purified compound **14**, detected at 260 nm.

Ac-K-ggcagtgtttcttg-Aeg(Azo)-K-CONH$_2$ (15)

Product (0.16 µmol, 6%) was obtained as a yellow solid. t_R = 13.70 min (Gradient II). HRMS-ESI (m/z): $[M+6H]^{6+}$ calcd for $C_{183}H_{239}N_{85}O_{52}$, 743.4781; found, 743.4793.

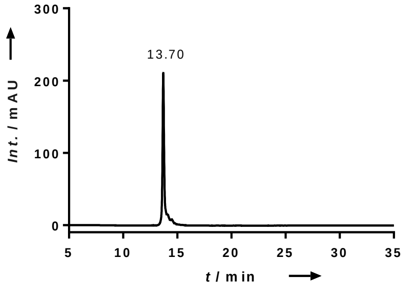

Figure S19: RP-HPLC chromatogram of purified compound **15**, detected at 260 nm.

Ac-K-ggc-Aeg(Azo)-gtgtttc-Aeg(Azo)-tgg-K-CONH$_2$ (16)

Product (0.16 µmol, 6%) was obtained as a yellow solid. t_R = 15.13 min (Gradient II). HRMS-ESI (m/z): [M+6H]$^{6+}$ calcd for $C_{189}H_{241}N_{85}O_{51}$, 753.1483; found, 753.1494.

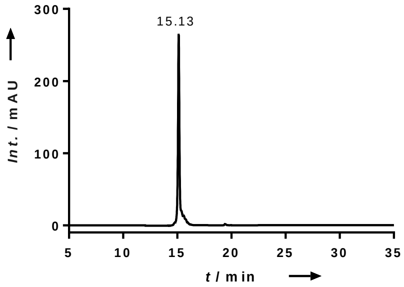

Figure S20: RP-HPLC chromatogram of purified compound **16**, detected at 260 nm.

Ac-K-gg-Aeg(Azo)-agtg-Aeg(Azo)-ttct-Aeg(Azo)-gg-K-CONH$_2$ (17)

Product (0.13 µmol, 5%) was obtained as a yellow solid. t_R = 16.84 min (Gradient II). HRMS-ESI (m/z): [M+6H]$^{6+}$ calcd for $C_{196}H_{243}N_{87}O_{48}$, 764.1545; found, 764.1556.

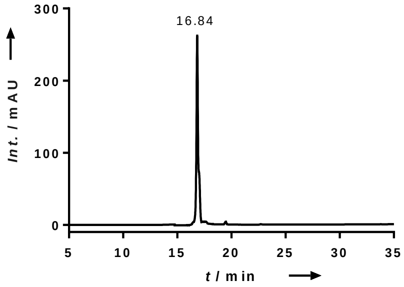

Figure S21: RP-HPLC chromatogram of purified compound **17**, detected at 260 nm.

Ac-K-ggcagtgtttcttgg-K-CONH$_2$ (18)

Product (0.30 µmol, 15%) was obtained as a white solid. t_R = 10.97 min (Gradient III). HRMS-ESI (m/z): [M+6H]$^{6+}$ calcd for $C_{177}H_{236}N_{88}O_{53}$, 740.6416; found, 740.6425.

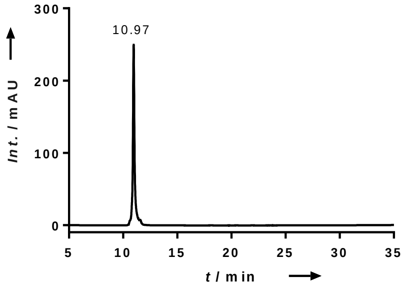

Figure S22: RP-HPLC chromatogram of purified compound **18**, detected at 260 nm.

Ac-K-tgagtgcgtctgttg-K-CONH$_2$ (19)

Product (0.38 µmol, 19%) was obtained as a white solid. t_R = 11.21 min (Gradient III). HRMS-ESI (m/z): $[M+6H]^{6+}$ calcd for $C_{177}H_{236}N_{88}O_{53}$, 740.6417; found, 740.6427.

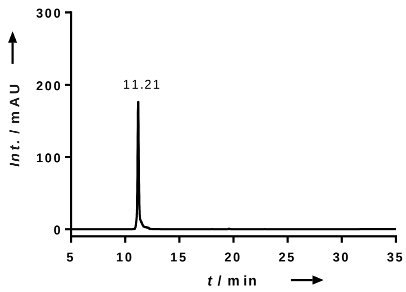

Figure S23: RP-HPLC chromatogram of purified compound **19**, detected at 260 nm.

Photoisomerization of photoswitchable PNAs

For the UV–vis characterization studies, solutions of corresponding PNA-photoswitch conjugates were prepared in phosphate buffer (10 mM NaH_2PO_4, 150 mM NaCl, pH 7.4) with a final concentration of 20 µM, the samples were placed in a 1400 µL quartz cuvette (Hellma Analytics (104F-QS)) with a pathlength of 1 cm. The cuvette was irradiated at the respective wavelengths, placed in the dark at room temperature or incubated in a 37 °C water bath for the indicated times, followed by measuring the absorbance spectra at room temperature. All determinations of each compound were repeated two times independently i.e. from two different stock solutions. For all spectra, the corresponding background signal was subtracted.

UV–vis characterization of PNA_{12}(*o*F_4Azo) conjugate 3

Isomerization

A sample containing **3** was irradiated for 2 min at 405 nm to ensure maximal conversion to the *trans*-state and then irradiated at 520 nm for different time intervals from 1 s to 5 min (1, 2, 5, 10, 15, 20, 30, 40, 60, 80, 100, 120, 180, 300 s). As shown in Figure S24A and S24C, after 3 min of continuous irradiation no significant spectroscopic changes could be observed. Afterwards, the sample was irradiated at the time intervals from 1 s to 30 s (1, 2, 5, 10, 15, 20, 30 s) at 405 nm (Figure S24B). As shown in Figure S24C, after 120 s irradiation at 520 nm, maximal *cis*-isomer ratio was reached. The maximal conversion from *cis* to *trans*-isomer was achieved after only 2 s.

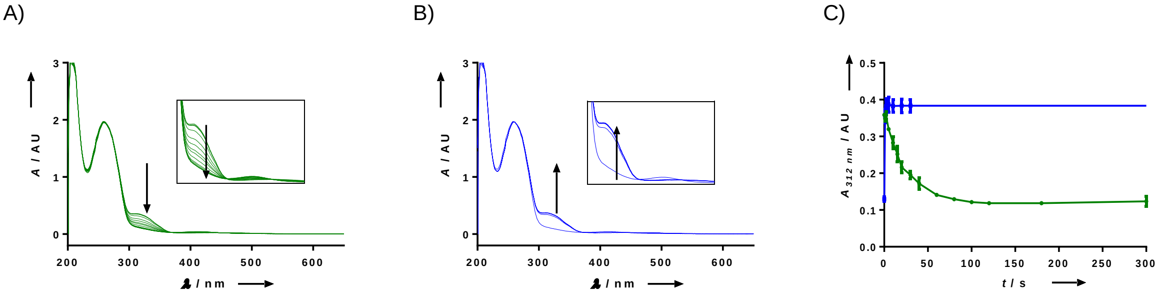

Figure S24: UV–vis spectra of 20 µM solution of compound **3** in phosphate buffer. A) 2 min irradiation at 405 nm followed by irradiation at 520 nm with superimposed time intervals (1, 2, 5, 10, 15, 20, 30, 40, 60, 80, 100, 120, 180, 300 s). B) Final irradiation conditions of A), followed by irradiation at 405 nm with superimposed time intervals (1, 2, 5, 10, 20, 30 s). C) Time-dependent absorbance change at 312 nm during irradiations. Blue: irradiation at 405 nm to obtain *trans* from *cis*; green: irradiation at 520 nm to obtain *cis* from *trans*. The values were measured in duplicates. UV–vis spectra were zoomed (285 nm to 500 nm) and inserted onto the corresponding spectra.

Reversibility

The reversibility of the isomerization of compound **3** was verified for at least 20 cycles; this conjugate did not show any photodegradation or photochemical fatigue.

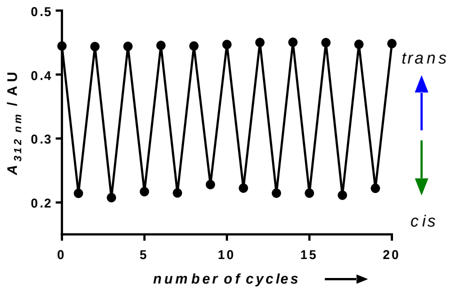

Figure S25: Reversible photochromism of a 20 µM solution of compound **3** in phosphate buffer upon alternating intervals of irradiation at 405 nm (1 min) to obtain *trans*-isomer and 520 nm (3 min) to obtain *cis*-isomer.

Thermal stability

A 2 µM sample containing **3** in phosphate buffer was irradiated at 405 nm for 2 min to obtain the maximal *trans*-isomer, the absorbance spectrum was measured once, and again after 24 h. As shown in Figure S26, there was no back-isomerization. The sample was irradiated at 520 nm for 5 min to obtain maximal *cis*-isomer, the absorbance was measured after the irradiation and after the incubation at room temperature as well as at 37 °C for 24 h each. No back-isomerization was observed neither.

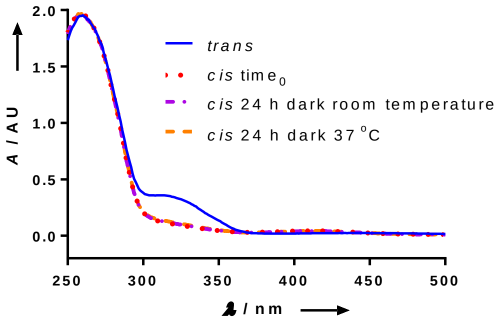

Figure S26: Thermal stability of a 20 µM solution of compound **3** in phosphate buffer upon irradiation at 405 nm (1 min) to obtain maximal *trans*-isomer and at 520 nm (3 min) to obtain maximal *cis*-isomer; followed by incubation in the dark at room temperature and 37 °C.

The stability was tested at different temperatures to ensure that no degradation of compound **3** occurred during the measurement of the melting temperatures. Solutions of compound **3** with a concentration of 20 µM in phosphate buffer were first irradiated at 520 nm for 5 min and 405 nm for 2 min, respectively, and then incubated for 10 min at various temperature from 20 °C to 90 °C. UV–vis spectra were measured to detect changes (Figure S27A and S27B). After each incubation, the sample was subjected to analytical RP-HPLC analysis to detect

degradation. After the incubation up to 90 °C no significant change of absorbance at 304 nm was observed. RP-HPLC chromatograms showed no degradation (Figure S27C).

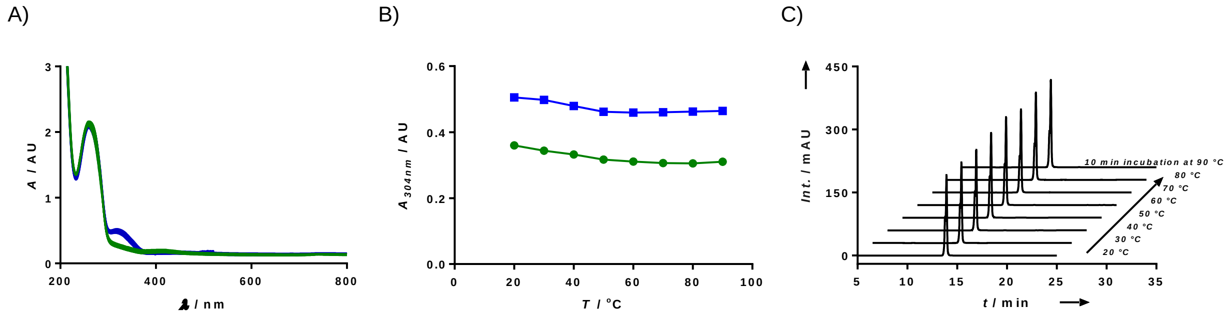

Figure S27: Thermal stability of 20 µM solutions of compound **3** in phosphate buffer upon irradiation at 520 nm (5 min) to obtain maximal *cis*-isomer and at 405 nm (2 min) to obtain maximal *trans*-isomer. Followed by incubation in the dark at various temperatures from 20 °C to 90 °C. The *trans*-isomer is shown in blue and the *cis*-isomer in green. A) UV–vis spectra of and B) Temperature dependent absorbance changes at 312 nm during irradiations. The values were measured in duplicates. C) HPLC chromatograms of PNA after incubation at various temperature (gradient II) detected at 260 nm.

Further the stability of compound **3** at 90 °C was tested. Samples with concentrations of 20 µM in phosphate buffer were irradiated at 520 nm for 5 min and 405 nm for 2 min, and afterwards heated to 90 °C, the UV–vis spectra were measured to detect any changes. The absorbance of the *cis*-isomer at 304 nm increased and reached its maxima after 8 h.

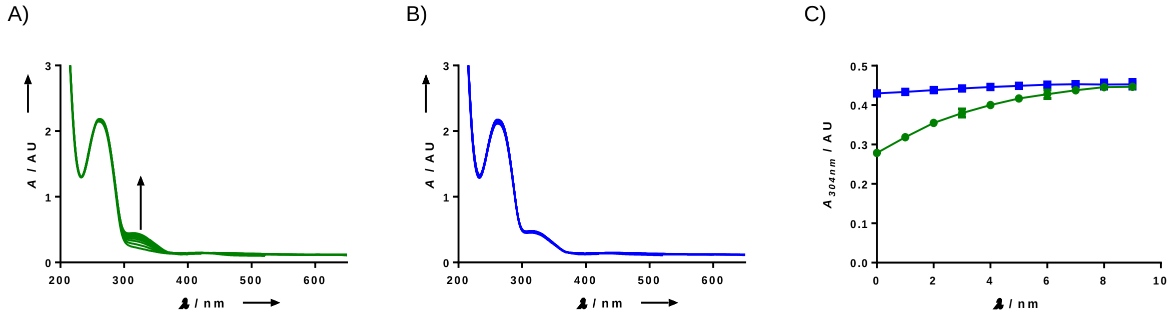

Figure S28: Thermal stability test of the isomers of compound **3** at 90 °C. A) *trans*-isomer. B) *cis*-isomer. C) Time dependent absorbance changes at 304 nm during incubation at 90 °C. Blue: *trans*-isomer; green: *cis*-isomer. The values were measured in duplicates.

RP-HPLC characterization of oF$_4$Azo conjugates

For the characterization of the photoisomerization of the compounds **1** and **3** via RP-HPLC measurements, a solution of the corresponding compounds with concentration of 20 µM each in phosphate buffer was irradiated for 2 min at 405 nm to obtain the maximum *trans*-isomer and for 5 min at 520 nm to obtain the maximum *cis*-isomer. The ratios of photostationary states reached upon irradiation were determined by integration of the peak areas in the RP-HPLC chromatograms at the isosbestic point of *o*F$_4$Azo (275 nm) with Agilent OpenLab software. Figure S29A shows the RP-HPLC chromatograms of **1** with *trans*/*cis*-ratio after irradiation, with t_R = 17.28 min corresponding to the *cis*-isomer and 17.78 min to the *trans*-isomer (Gradient III).

Figure S29B shows the RP-HPLC chromatograms of **3** with *trans*/*cis*-ratio after irradiation, with t_R = 19.30 min corresponding to the *trans*-isomer and 19.65 min to the *cis*-isomer (Gradient I).

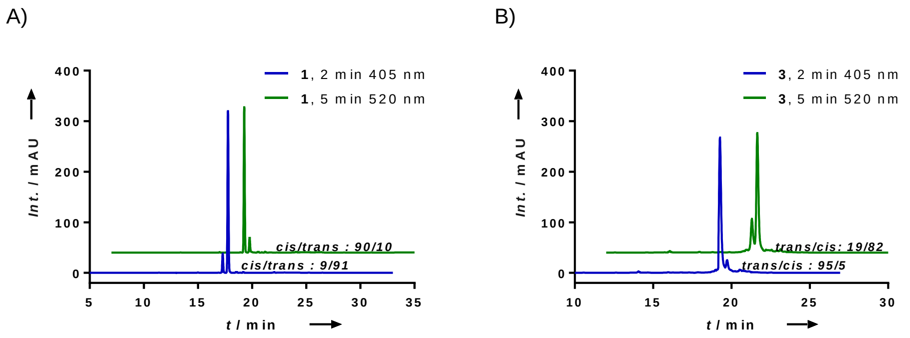

Figure S29: RP-HPLC chromatograms of A) compound **1** and B) compound **3** after irradiation at 405 nm for 2 min (blue) and 520 nm for 5 min (green) respectively and corresponding *trans*/*cis*-ratio. Detected at isosbestic point of *o*F_4Azo: 275 nm.

UV–vis characterization of PNA12(HTI) conjugate 4

Isomerization

The isomerization of HTI could be achieved under the irradiation at 405 nm for the *trans*-isomer, the *cis*-isomer could be obtained either upon irradiation at 520 nm or when heated.[7][8]

A 20 µM solution of compound **4** in phosphate buffer was irradiated for 2 min at 405 nm to ensure maximal conversion to the *trans* state and then irradiated at 520 nm for different time intervals from 10 s to 30 min (10, 20, 30 s, 1, 2, 3, 4, 5, 7, 10, 15, 20, 30 min) (Figure S30A). Afterwards the sample was irradiated at different time intervals from 1 s to 1 min (1, 2, 5, 10, 20, 30, 60 s) at 405 nm (Figure S30B). As shown in Figure S30C, after 10 min irradiation at 520 nm, maximal *cis*-isomer ratio was reached. The maximal conversion from *cis*- to *trans*-isomer was achieved after only 30 s upon irradiation at 405 nm.

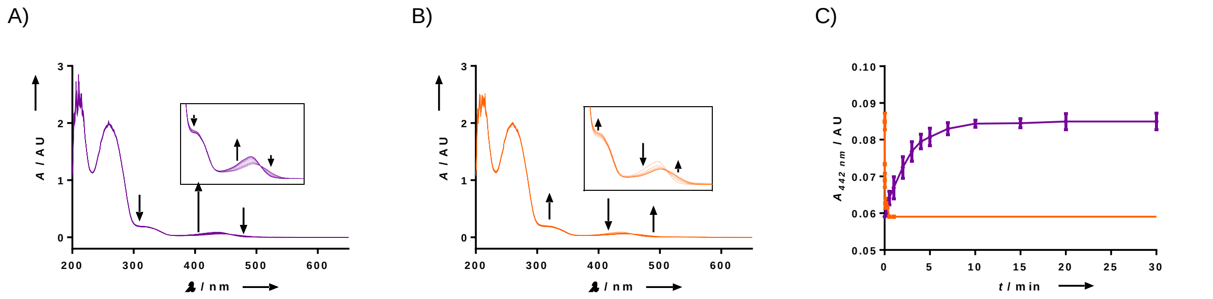

Figure S30: UV–vis spectra of 20 µM solutions of compound **4** in phosphate buffer. A) 5 min irradiation at 405 nm followed by irradiation at 520 nm with superimposed time intervals (10, 20, 30 s, 1, 2, 3, 4, 5, 7, 10, 15, 20, 30 min). B) Final irradiation conditions of A), followed by irradiation at 405 nm with superimposed time intervals (1, 2, 5, 10, 20, 30, 60 s). C) Time dependent absorbance changes at 442 nm during irradiations. Orange: irradiation at 405 nm to obtain *trans* from *cis*; Purple: irradiation at 520 nm to obtain *cis* from *trans*. The measurements were performed in duplicate. UV–vis spectra were zoomed (285 nm to 550 nm) and inserted onto the corresponding spectra.

Reversibility

The reversibility of PNA12(HTI) (**3**) switching was analyzed, after 20 cycles.

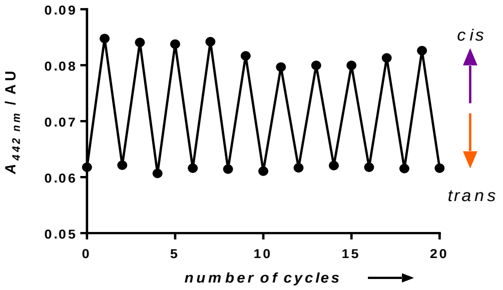

Figure S31: Reversible photochromism of PNA12(HTI) upon alternating intervals of irradiation at 405 nm (2 min) to obtain *trans*-isomer and 520 nm (5 min) to obtain *cis*-isomer. 20 µM of **4** in phosphate buffer.

Stability

The *cis*-isomer of HTI is thermally more stable, the *trans*-isomer could reconvert at room temperature to the *cis*-isomer within hours.[9] A 20 µM sample of compound **4** in phosphate buffer was irradiated at 405 nm for 2 min to obtain the maximum *trans*-isomer, absorbance spectra were measured every 15 min at room temperature on the time scale of 10 hours. As shown in Figure S32, the significant absorbance at 442 nm of the *cis*-isomer was increasing with time and reached maximum after 6 h.

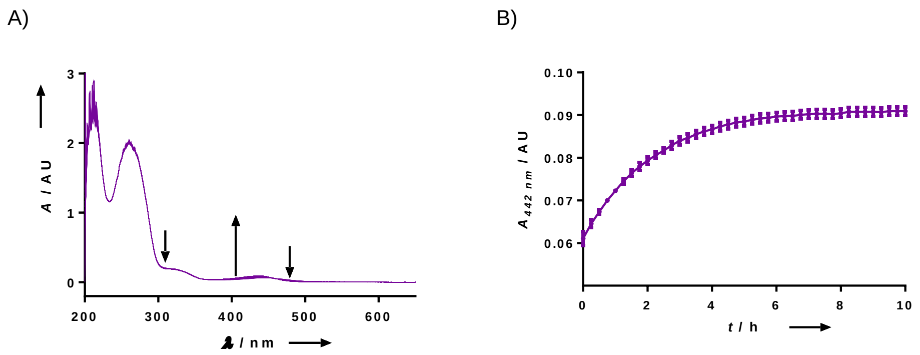

Figure S32: Thermal stability of a 20 µM solution of **4** in phosphate buffer irradiation at 405 nm (2 min) to obtain the maximal *trans*-isomer ratio was followed by incubation in the dark at room temperature, UV–vis spectra were collected every 15 min.

RP-HPLC characterization of HTI conjugates

For the characterization of the photoisomerization of compounds **2** and **4** via RP-HPLC, 20 µM solutions of the corresponding compounds in phosphate buffer were irradiated for 2 min at 405 nm to obtain the maximum *trans*-isomer, and for 10 min at 520 nm to obtain the maximum *cis*-isomer. The ratios of photostationary states reached upon irradiation were determined by integration of the peak areas in the RP-HPLC chromatograms at the isosbestic point of HTI (275 nm). Figure S33A shows the RP-HPLC chromatograms with *trans*/*cis*-ratios after irradiation of compound **2**, t_R = 22.70 min accounts for the *cis*-isomer and 23.08 min for the *trans*-isomer (Gradient III). Figure S33B shows the RP-HPLC chromatograms with *trans*/*cis*-ratios after irradiation of compound **4**, t_R = 18.73 min accounts for the *cis*-isomer and 19.55 min accounts for the *trans*-isomer (Gradient IV).

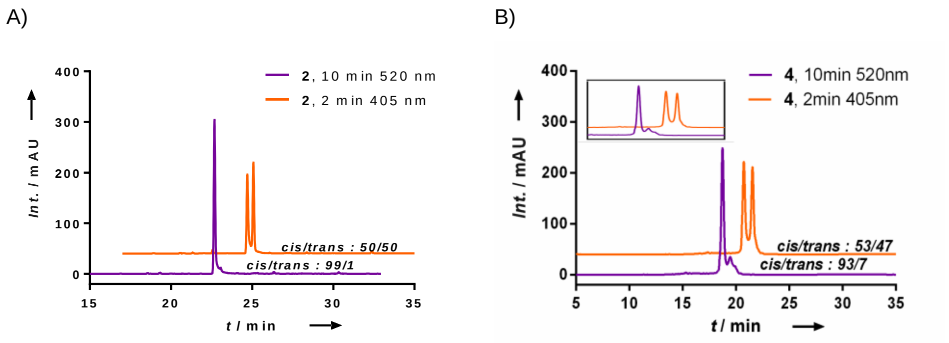

Figure S33: RP-HPLC chromatograms of A) compound **2** and B) compound **4** after irradiation at 405 nm for 2 min and 520 nm for 10 min and their corresponding *trans*/*cis*-ratio. Detected at isosbestic point of HTI: 275 nm. RP-HPLC chromatograph was zoomed (15 to 25 min) and inserted onto the chromatograph.

UV–vis characterization of PNA15(Azo) conjugate 14

Thermal stability

The stability was tested at different temperatures to ensure that no degradation of compound **14** occurrs during the measurements of the melting temperature. 25 μM solutions of the compound **14** in phosphate buffer were first irradiated at 365 nm for 1 h to obtain the maximum *cis*-form and at 430 nm for 30 min to obtain the maximum *trans*-form, then the samples were incubated for 10 min at various temperatures from 20 °C to 90 °C, the UV–vis spectra were recorded to detect changes.

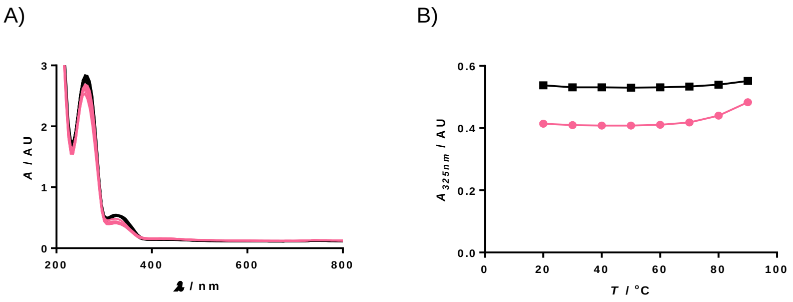

Figure S34: Thermal stability test of 25 μM solutions of compound **14** in phosphate buffer upon irradiation at 365 nm (1 h) to obtain maximal *cis*-isomer and at 430 nm (30 min) to obtain maximal *trans*-isomer. Followed by incubation in the dark at various temperatures from 20 °C to 90 °C respectively. A) UV–vis spectra of *trans*-isomer: black; *cis*-isomer: pink. B) Temperature dependent absorbance changes at 312 nm during irradiations. Black: *trans*-isomer; pink: *cis*-isomer.

The stability of the compound **14** at 90 °C was tested. The same samples as for the temperature-dependent stability study were incubated further at 90 °C, UV–vis spectra were measured superimposed time intervals (10, 20, 30, 60 min) to detect changes. The absorbance characteristic for the *cis* form at 325 nm increased over time and reached its maximum after 30 min.

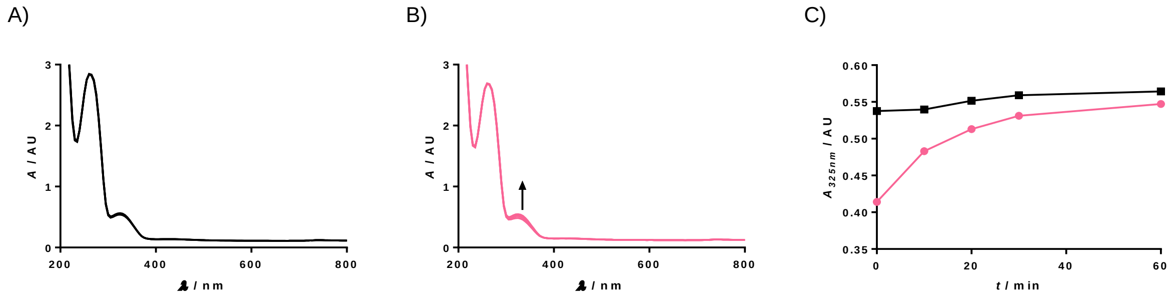

Figure S35: Thermal stability test of 25 μM solutions of isomers of compound **14** at 90 °C. UV–vis spectra for A) the *trans*-isomer (30 min irradiation at 430 nm); B) the *cis*-isomer (1 h irradiation at 365 nm) followed time intervals (10, 20, 30, 60 min). C) Time-dependent absorbance changes at 325 nm during incubation at 90 °C. Black: *trans*-isomer; pink: *cis*-isomer.

RP-HPLC characterization of PNA15(Azo) conjugate 14

For the characterization of the photoisomerization of compound **14** via RP-HPLC measurements, 25 µM solutions of the compound **14** in phosphate buffer were irradiated at 365 nm for 1 h to obtain the *cis*-form or at 430 nm for 30 min to obtain the *trans*-form. The photostationary states reached upon irradiation were determined by integration of the peak areas in the RP-HPLC chromatogram at the isosbestic wavelength of Azo (275 nm). Figure S36 shows the RP-HPLC chromatograms of compound **14** with *trans*/*cis*-ratios after irradiation, t_R = 16.77 min corresponds to the *cis*-isomer and 17.97 min to the *trans*-isomer (Gradient I).

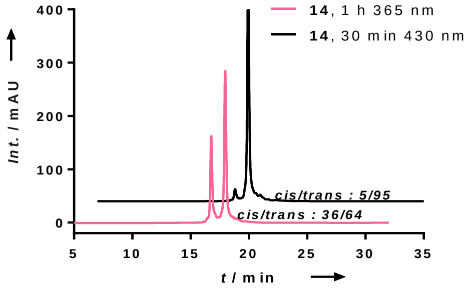

Figure S36: RP-HPLC chromatograms of compound **14** after irradiation at 365 nm for 1 h and 430 nm (pink) and for 30 min and corresponding *trans*/*cis*-ratios. Detected at isosbestic point of PNA_{15}(Azo): 275 nm.

Studies of PNA/DNA hybridization

UV Melting curves

UV melting curves were determined at the concentrations of 1 µM of DNA as well as PNA in phosphate buffer (10 mM NaH_2PO_4, 150 mM NaCl, pH 7.4). The concentrations of stock solutions of DNA (G/A/C/T) and PNA (g/a/c/t) without modifications in Milli-Q water were determined by measuring the UV absorbance at 260 nm. The stock concentrations of DNAs were calculated by applying the molar extinction coefficients provided by the commercial supplier IDT (USA). The stock concentrations of PNA were calculated utilizing the molar extinction coefficient obtained by the following extinction coefficients for the nucleobases: g = 12000; a = 15200; c = 7050; *t* = 8400 [$M^{-1}cm^{-1}$] applied in the following equation:[10]

$$\varepsilon_{260\ nm} = \{(8.8 \times n_T) + (7.3 \times n_C) + (11.7 \times n_G) + (15.4 \times n_A)\} \times 0.9 \times 10^3\ M^{-1}cm^{-1}$$

Where n represents the frequency of occurrence of the bases in the conjugate.

The concentration of photoswitch modified PNAs were determined by applying the following molar extinction coefficients with Lambert-Beer law.

Table S1: Molar extinction coefficient of photoswitches.

Compounds	solvent	*λ* [nm]	*ε* [$M^{-1}cm^{-1}$]
***o*F$_4$Azo**	MeCN	305	19000[3]
**Azo**	DMSO	338	16170[11]
**HTI**	MeCN	320	14100[12]

All melting curves were measured in phosphate buffer (10 mM NaH_2PO_4, 150 mM NaCl, pH = 7.4) at a heating rate of 2 °C/min. In order to obtain maximal isomer, the melting temperature PNA(*o*F$_4$Azo) was measured without pre-hybridization due to the difficult photoisomerization of PNA(*o*F$_4$Azo)/DNA duplex. As the *cis*-isomer of PNA(Azo) is not thermally stable, its photoisomerization was performed after the hybridization,

Sample solutions of compounds **6**-**11** (*cis*/*trans)*-isomer conversion was achieved by irradiation of the sample at room temperature with 405 nm/520 nm LED for *o*F$_4$Azo (*cis*/*trans*) and HTI (*cis*/*trans*), illumination at 430 nm for Azo (*trans*). Samples were kept in the dark. After isomerization to the corresponding isomers, the complementary DNA (5'-GTG AGC CAA GAA ACA CTG CCT-3') was added in a 1:1 ration and the solutions mixed in the dark under red light while the isomers are stable under red light. Duplex forming DNAs were prepared by mixing respective DNAs (11-mer: 5'-FAM-CAG TGT TTC TTA TGC-3', 15-mer: 5'-FAM-TGG CAG TGT TTC TTG G-3') and the complementary DNA (5'-ATG TGA GCC AAG AAA CAC TGC CTT C-BHQ-3') in a 1:1 ration. The UV melting curves were measured from 20 °C to

95 °C at 260 nm for 3 cycles without pre-hybridization. Melting curves were collected from the second and third cycles of the measurements.

Trans-isomers of compounds **12–17** were prepared by using the method described above. *Cis*-isomers of compounds **12–17** were prepared by mixing respective PNAs and the complementary DNA (5'-GTG AGC CAA GAA ACA CTG CCT-3'), heating the sample to 90 °C and keeping them at this temperature for 15 min before cooling to room temperature during 3 h. Isomer conversion was achieved by irradiation of the hybridized sample at room temperature at 365 nm for 1 h for Azo (*cis*). Likewise the UV melting curve was measured from 20 °C to 95 °C at 260 nm in 2 °C/min steps.

All melting temperatures were determined from the first derivative of the absorbance temperature trace using OriginPro 2016 software. All determinations were repeated two times independently i.e. from two different stock solutions. For all data, the corresponding background signal was subtracted, and the melting curves were normalized.

The sequences of all employed PNAs and DNAs are summarized in Table S2.

Table S2: Sequences of probe and target strands.

Compounds	Sequence
**3**	Ac-ggcagAeg(*o*F$_4$Azo)gtttct-CONH$_2$
**4**	Ac-ggcagAeg(HTI)gtttct-CONH$_2$
**5**	Ac-ggcagAeg(Ac)gtttct-CONH$_2$
**6**	Ac-*Lys*-Aeg(*o*F$_4$Azo)gcagtgtttcttgg-*Lys*-CONH$_2$
**7**	Ac-*Lys*-ggAeg(*o*F$_4$Azo)agtgtttcttgg-*Lys*-CONH$_2$
**8**	Ac-*Lys*-ggcagtAeg(*o*F$_4$Azo)tttcttgg-*Lys*-CONH$_2$
**9**	Ac-*Lys*-ggcagtgtttcttgAeg(*o*F$_4$Azo)-*Lys*-CONH$_2$
**10**	Ac-*Lys*-ggcAeg(*o*F$_4$Azo)gtgtttcAeg(*o*F$_4$Azo)tgg-*Lys*-CONH$_2$
**11**	Ac-*Lys*-ggAeg(*o*F$_4$Azo)agtgAeg(*o*F$_4$Azo)ttctAeg(*o*F$_4$Azo)gg-*Lys*-CONH$_2$
**12**	Ac-*Lys*-Aeg(Azo)-gcagtgtttcttgg-*Lys*-CONH$_2$
**13**	Ac-*Lys*-ggAeg(Azo)agtgtttcttgg-*Lys*-CONH$_2$
**14**	Ac-*Lys*-ggcagtAeg(Azo)tttcttgg-*Lys*-CONH$_2$
**15**	Ac-*Lys*-ggcagtgtttcttgAeg(Azo)-*Lys*-CONH$_2$
**16**	Ac-*Lys*-ggcAeg(Azo)gtgtttcAeg(Azo)tgg-*Lys*-CONH$_2$
**17**	Ac-*Lys*-ggAeg(Azo)agtgAeg(Azo)ttctAeg(Azo)gg-*Lys*-CONH$_2$
**18**	Ac-*Lys*-ggcagtgtttcttgg-*Lys*-CONH$_2$
**19**	Ac-*Lys*-tgagtgcgtctgttg-*Lys*-CONH$_2$
**complementary DNA**	5'-GTG AGC CAA GAA ACA CTG CCT-3'
**15-mer dsDNA**	5'-**FAM**-TGG CAG TGT TTC TTG G-3'<br>5'-ATG TGA GCC AAG AAA CAC TGC CTT C-**BHQ**-3'
**11-mer dsDNA**	5'-**FAM**-CAG TGT TTC TTA TGC-3'<br>5'-ATG TGA GCC AAG AAA CAC TGC CTT C-**BHQ**-3'

The absorbance differentials at 260 nm were plotted against temperature and normalized. Obtained UV melting curves and calculated first derivative curves for the studied compounds are given in Figures S37 to Figure S45.

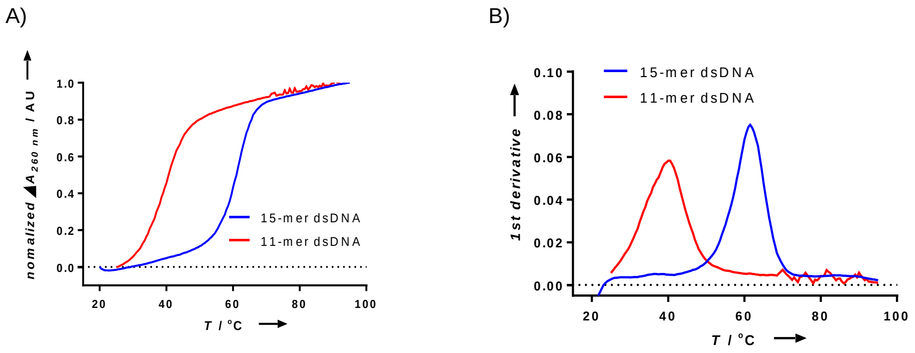

Figure S37: A) UV melting curves of **15-mer BHQ/FAM-dsDNA** (blue) and **11-mer** (red). Differential absorbance at 260 nm versus temperature. B) Corresponding first derivative curves. T_M calculated for **15-mer** dsDNA: 61.5 °C; **11-mer** dsDNA: 39.9 °C.

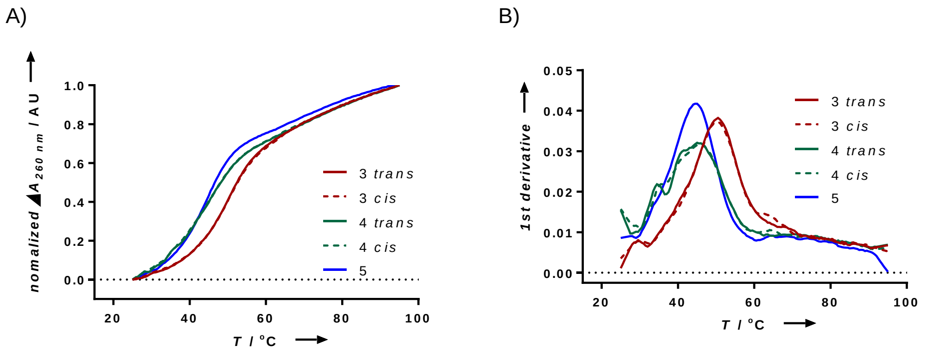

Figure S38: A) UV melting curves of compounds **3** (red) and **4** (green), solid line for *trans*- and dashed line for *cis*-isomers as well as **5** (blue) with complementary DNA. Differential absorbance at 260 nm versus temperature. B): Corresponding first derivative curves.

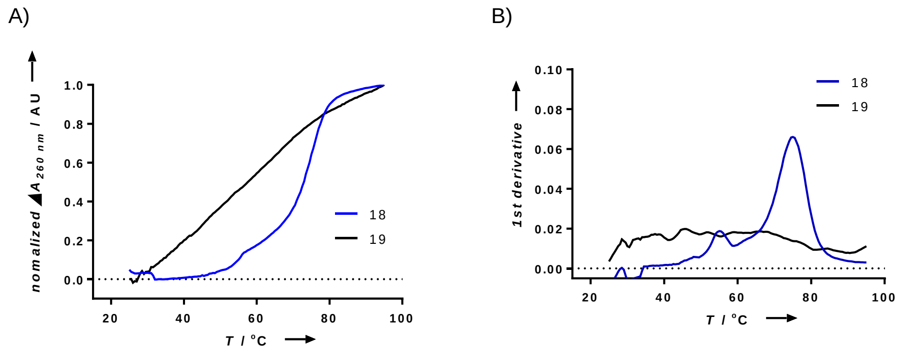

Figure S39: A) UV melting curves of compounds **18** (blue) and **19** (black) with complementary DNA. Differential absorbance at 260 nm versus temperature. B) Corresponding first derivative curves.

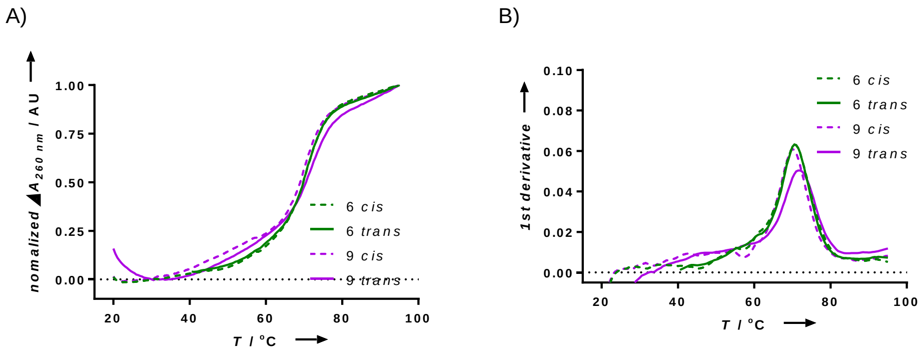

Figure S40: A) UV melting curves of compounds **6** (green) and **9** (pink) with complementary DNA, solid line for *trans*-isomers and dashed line for *cis*-isomers. Differential absorbance at 260 nm versus temperature. B) Corresponding first derivative curves.

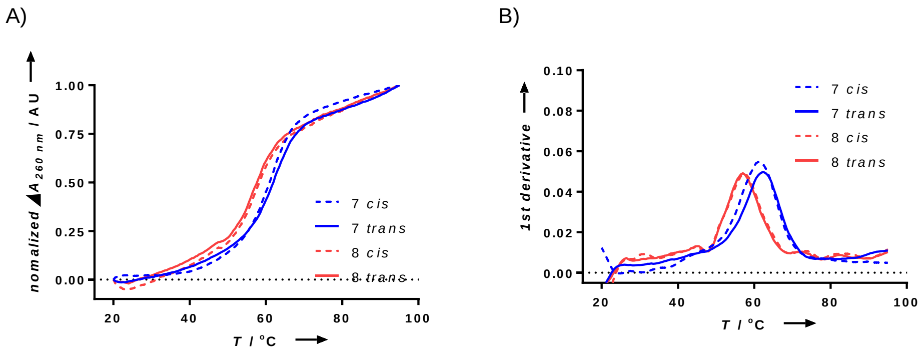

Figure S41: A) UV melting curves of compounds **7** (blue) and **8** (red) with complementary DNA, solid line for *trans*-somers and dashed line for *cis*-isomers. Differential absorbance at 260 nm versus temperature. B) Corresponding first derivative curves.

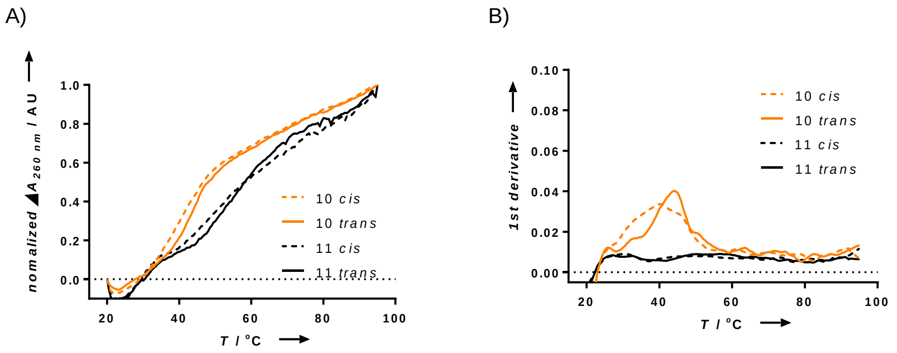

Figure S42: A) UV melting curves of compounds **10** (orange) and **11** (black) with complementary DNA, solid line for *trans*-isomers and dashed line for *cis*-isomers. Differential absorbance at 260 nm versus temperature. B) Corresponding first derivative curves.

A) B)

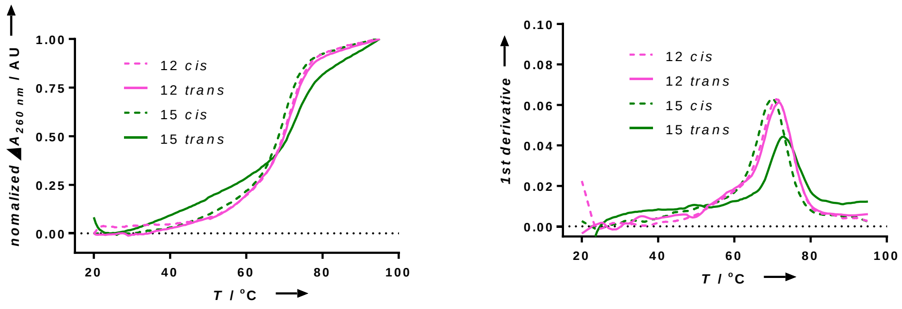

Figure S43: A) UV melting curves of compounds **12** (pink) and **15** (green) with complementary DNA, solid line for *trans*-isomers and dashed line for *cis*-isomers. Differential absorbance at 260 nm versus temperature. B) Corresponding first derivative curves.

A) B)

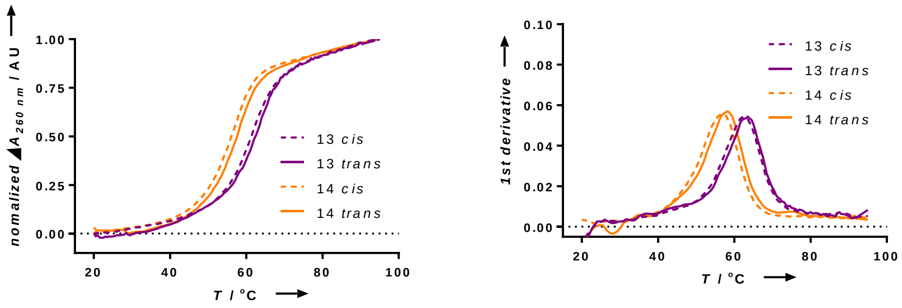

Figure S44: A) UV melting curves of compounds **13** (pink) and **14** (green) with complementary DNA, solid line for *trans*-isomers and dashed line for *cis*-isomers. Differential absorbance at 260 nm versus temperature. B) Corresponding first derivative curves.

A) B)

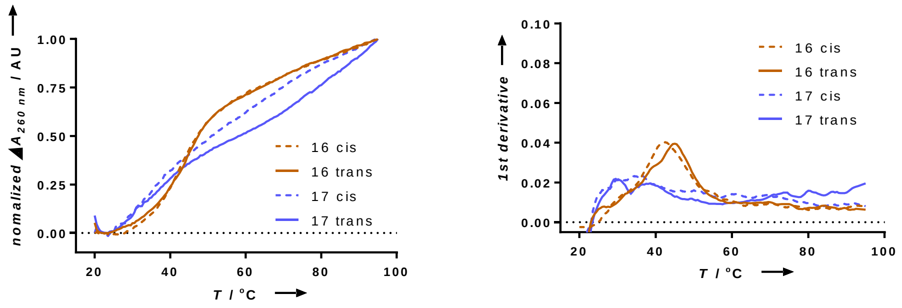

Figure S45: A) UV melting curves of compounds **16** (kaki) and **17** (lavender) with complementary DNA, solid line for *trans*-isomers and dashed line for *cis*-isomers. Differential absorbance at 260 nm versus temperature. B): Corresponding first derivative curves.

Displacement experiment

First, the possible quenching effect of studied PNAs on complementary FAM-ssDNA (15-mer: 5’-FAM-TGG CAG TGT TTC TTG G-3’) was scanned at a concentration of 0.75 µM for the FAM-ssDNA in presence of 2 equiv of corresponding PNAs in PBS buffer (140 mM NaCl, 10 mM Na_2HPO_4, 2.7 mM KCl, 1.8 mM KH_2PO_4, pH 8.0) and compared with BHQ/FAM-dsDNA. Fluorescence intensity was measured every 10 min during 8 h at 37 °C. Every different isomer of each compound was measured in triplicate during one measurement and all determinations were performed in independent duplicates, i.e., from two different stock solutions. For all values, the corresponding buffer background signal was subtracted.

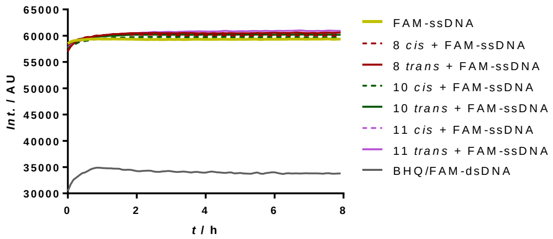

Figure S46: Time-dependent fluorescence changes; yellow: FAM-ssDNA, gray: quenched control: BHQ/FAM-dsDNA, FAM ssDNA in presence of compounds, red: **8**; green: **10**; pink: **11**. Fluorescence intensities were measured every 10 min during 8 h at 37 °C.

Displacement experiments were carried out at concentrations of 0.75 µM DNAs with 2 equiv of corresponding PNA_{15}(*o*F_4Azo)s **6**-**11**, unmodified PNA **18** and scrambled PNA **19** in PBS buffer (140 mM NaCl, 10 mM Na_2HPO_4, 2.7 mM KCl, 1.8 mM KH_2PO_4, pH 8.0). The duplex DNA was prepared by mixing the FAM-ssDNAs (15-mer: 5’-FAM-TGG CAG TGT TTC TTG G-3’; 11-mer: 5’-FAM-CAG TGT TTC TTA TGC-3’) and BHQ-ssDNA (5’-ATG TGA GCC AAG AAA CAC TGC CTT C-BHQ-3’) in a 1:1 ratio in the same buffer and heating the mixture at 95 °C for 15 min, and afterwards this was slowly cooled down to room temperature within 3 h.

For each well, 100 µL dsDNA stock was mixed with 2 µL of the corresponding 37.5 µM PNA stock solution and covered with 100 µL paraffin oil to avoid evaporation. The preparation of the samples was always done in the dark under red light to avoid any back isomerization. Fluorescence intensities were measured immediately after the preparation, every 5 min during 8 h at 37 °C for 15-mer BHQ/FAM-dsDNA (5’-FAM-TGG CAG TGT TTC TTG G-3’ / 5’-ATG TGA GCC AAG AAA CAC TGC CTT C-BHQ-3’) and during 12 h at 30 °C for 11-mer BHQ/FAM-dsDNA (5’-FAM-CAG TGT TTC TTA TGC-3’ / 5’-ATG TGA GCC AAG AAA CAC TGC CTT C-BHQ-3’) respectively. Every different isomers of each compound was measured in triplicate during one measurement and all determinations were performed in independent duplicates i.e. from two different stock solutions.

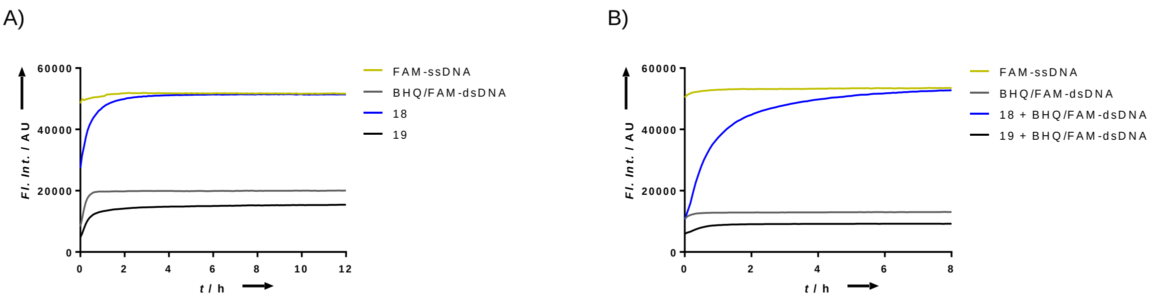

Figure S47: Time dependent fluorescence changes at 520 nm, yellow: fluorescent-labelled ssDNA, gray: quenched BHQ/FAM-dsDNA, blue: quenched BHQ/FAM-dsDNA in present of compound **18** and black: quenched BHQ/FAM-dsDNA in presence of compound **19**. Fluorescence intensities were measured every 5 min during A) 12 h at 30 °C with 11-mer dsDNA and B) 8 h at 37 °C with 15-mer dsDNA.

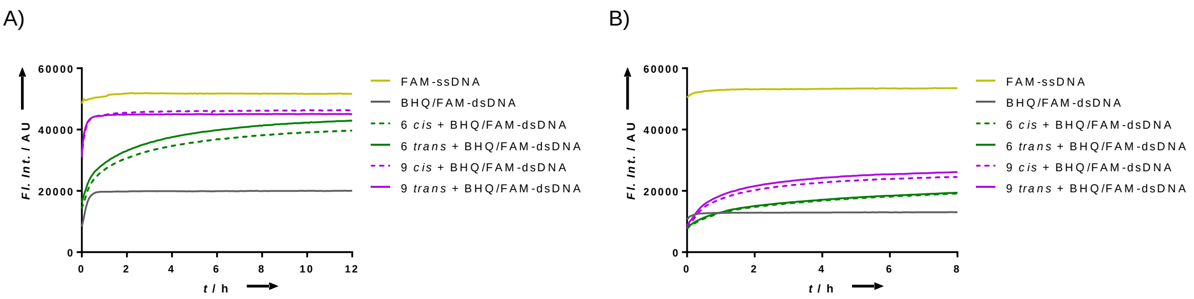

Figure S48: Time-dependent fluorescence changes at 520 nm, yellow: fluorescent-labelled ssDNA, gray: quenched BHQ/FAM-dsDNA, green: quenched BHQ/FAM-dsDNA in present of compound **6** and pink: quenched BHQ/FAM-dsDNA in present of compound **9**, solid lines for both *trans*-isomers and controls; dashed lines for *cis*-isomers. Fluorescence intensities were measured every 5 min during A) 12 h at 30 °C with 11-mer dsDNA and B) 8 h at 37 °C with 15-mer dsDNA.

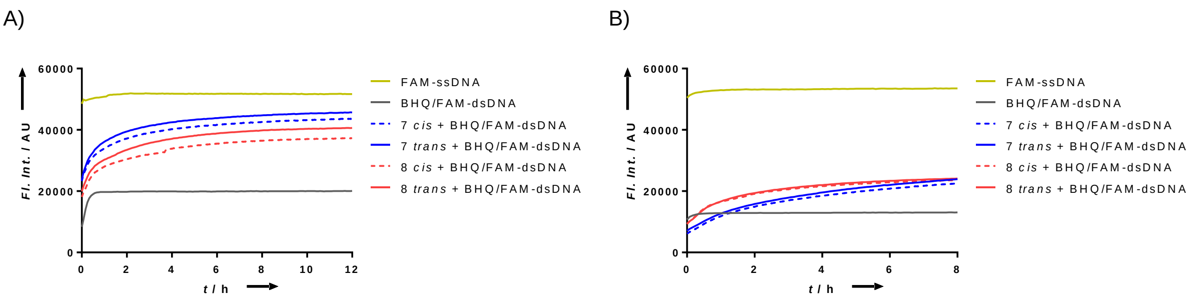

Figure S49: Time dependent fluorescence changes at 520 nm, yellow: fluorescent-labelled ssDNA, gray: quenched dsDNA, blue: quenched BHQ/FAM-dsDNA in present of compound **7** and red: quenched BHQ/FAM-dsDNA in present of compound **8**, solid lines for both *trans*-isomers and controls; dashed lines for *cis*-isomers. Fluorescence intensities were measured every 5 min during A) 12 h at 30 °C with 11-mer dsDNA and B) 8 h at 37 °C with 15-mer dsDNA.

A) B)

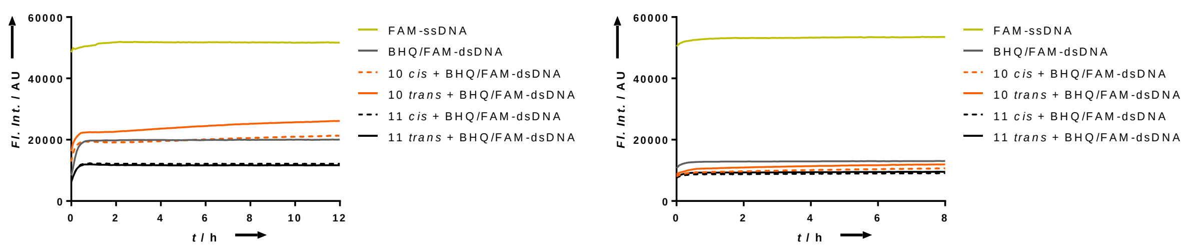

Figure S50: Time dependent fluorescence changes at 520 nm, yellow: fluorescent-labelled ssDNA, gray: quenched dsDNA, orange: quenched BHQ/FAM-dsDNA in present of compound **10** and black: quenched BHQ/FAM-dsDNA in present of compound **11**, solid lines for both *trans*-isomers and controls; dashed lines for *cis*-isomers. Fluorescence intensities were measured every 5 min during A) 12 h at 30 °C with 11-mer dsDNA and B) 8 h at 37 °C with 15-mer dsDNA.

Time dependent fluorescence changes at 37 °C of PNA_{15}(*o*F_4Azo) **6**, **7**, **8** and **9**, **10**, **11**, **18**, **19** were plotted as in the Fig 5 in the manuscript to facilitate comparison. Percentages were calculated according the endpoint fluorescence intensity values of each compound and FAM-ssDNA considering FAM-ssDNA as 100%.

A) B)

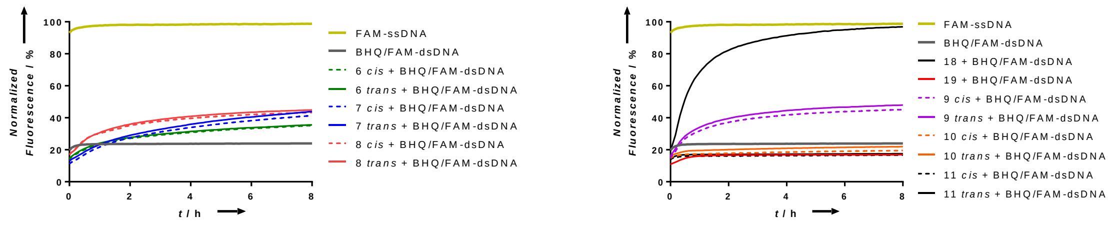

Figure S51: Time-dependent fluorescence signals of from two independent experiments at 520 nm of 0.75 µM FAM/BHQ-dsDNA 15-mer solutions in PBS buffer (140 mM NaCl, 10 mM Na_2HPO_4, 2.7 mM KCl, 1.8 mM KH_2PO_4, pH 8.0) with 2 equiv of the corresponding PNAs **6**-**11, 18** and **19** placed in 96-well plates and cover with paraffin oil; fluorescence intensities were measured every 5 min during 12 h at 30 °C; PNA code: **6**: green, **7**: blue, **8**: coral, **9**: pink, **10**: orange, **11**: gray, **18**: black and **19**: red; solid line for *trans*-isomers and dashed line for *cis*-isomers. Black lines controls: solid: FAM-ssDNA without PNA (mustard) and under the same conditions; BHQ/FAM-dsDNA (dark grey).

Table S3 compares isomer differences between those obtained in the melting temperature experiments and fluorescence displacement assays for the PNA probes **6-9**.

Table S3: Isomer-difference correlation between melting temperature experiments and fluorescence displacement assays for PNA/DNA 15mer probes.

Compounds	Δ T_M / °C	Δ endpoint fluorescence intensity / %
**6**	0.2	0.5
**7**	1.0	2.8
**8**	0.3	1.0
**9**	1.7	2.9

CD Experiments

For the characterization of the binding between our probes and DNA the concentrations of the PNAs **9**, **18** and **19**, compound **1** as well as complementary ssDNA (5’-GTG AGC CAA GAA ACA CTG CCT-3’) were determined as described in the UV melting curves section. Solutions of 5 µM compound, ssDNA or 1:1 ratio of PNA and ssDNA in phosphate buffer (10 mM NaH_2PO_4, 150 mM NaCl, pH 7.4) were prepared in microcentrifuge tubes from 100 mM stocks in phosphate buffer, transferred to a CD cuvette. Isomer conversion was achieved by irradiation of the sample at room temperature at 405 nm for 5 min for *trans-* or for 10 min at 520 nm for the *cis*-isomer. Samples were kept in the dark and the measurements were performed in the absence of light. Spectra were buffer subtracted and display the mean out of two independent measurements. The final spectra were smoothed with a fourth order Savitsky–Golay smoothing algorithm [13] using a smoothing window of 25 data points. Figure S52 shows an example of the dataset for the 1:1 ratio of PNA **18** and complementary DNA before and after smoothing.

A) B)

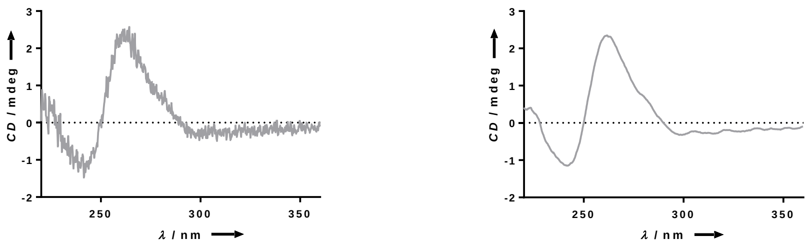

Figure S52: Example of smoothing of CD signals of 5 µM **18**/ssDNA solution in phosphate buffer (10 mM NaH_2PO_4, 150 mM NaCl, pH 7.4). A) displays the buffer subtracted mean of two independent experiments and B) the fourth order smoothing of 25 neighboring data points of A).

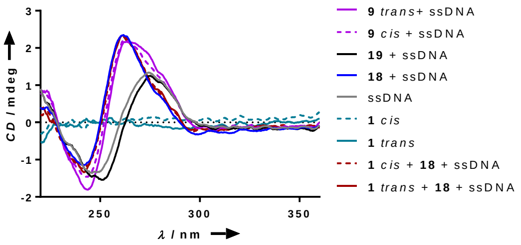

Figure S53: Smoothed mean value CD spectra of two independent experiments of ssDNA alone and in presence of *o*F_4Azo-PNA **9** (purple), unmodified target-PNA **18** (blue) or unmodified-scramble PNA **19** (black) as well as PNA **18** and complementary DNA in presence of compound **1** (burgundy) as well as of compound **1** alone (aqua); solid lines for both *trans*-isomers and controls; dashed lines for *cis*-isomers.

UV–vis Experiments

Further we performed UV-vis measurements of PNAs **9**, **18** and **19** in the presence and absence of complementary ssDNA (5'-GTG AGC CAA GAA ACA CTG CCT-3'). Samples were prepared as described for the CD experiments but at 2 µM concentration. Additional pre-hybridized samples were prepared. For the pre-hybridization samples containing 2 µM of the respective PNA and the complementary ssDNA were heated to 95 °C in a Thermomixer (Eppendorf, Germany) without shaking and kept at this temperature for 15 min before allowing to cool to room temperature during 3 h in the thermoblock. Spectra are buffer subtracted and show the mean out of two independent measurements.

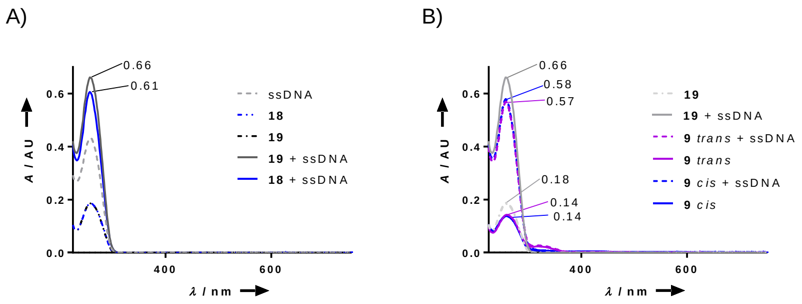

Figure S54: Comparison of UV-vis spectra for the characterization of PNA/DNA hybridization. ssDNA (grey), unmodified target-PNA **18** (blue) or unmodified-scramble PNA **19** (black). A) dashed lines for single strands and solid lines for samples containing equimolar amounts of PNA and DNA. B) Evaluation of hyperchromicity of the *cis*- (blue) and *trans-isomer* (purple) of **9** in the presence of ssDNA compared to unmodified-scramble PNA **19** (grey).

Isomer conversion was achieved by irradiation of the sample at room temperature at 520 nm for 10 min for *cis* or for 5 min at 405 nm for *trans*. Samples were kept in the dark and the measurements were performed in the absence of light.

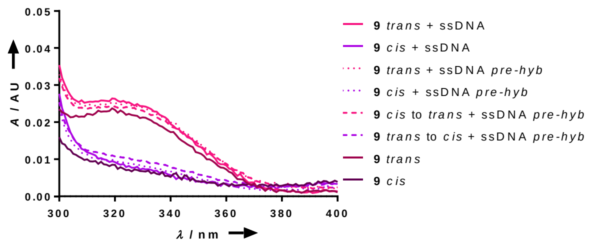

Figure S55: Zoom of UV-vis spectra evaluating changes in isomerization efficiency between pre-hybridized and non-prehybridized samples of compound **9** (*cis* purple and *trans* pink) and ssDNA. Dotted lines for prehybridized samples (dashed after switching) and solid lines for samples containing equimolar amounts of PNA and DNA. Dark lines represent isomers of compound **9** alone.

References

[1] G. R. Fulmer, A. J. M. Miller, N. H. Sherden, H. E. Gottlieb, A. Nudelman, B. M. Stoltz, J. E. Bercaw, K. I. Goldberg, R. Gan, H. Apiezon, *Organometallics* **2010**, *29*, 2176–2179.

[2] W. H. B. Clement Appiah, Georg Woltersdorf, *Polym. Chem.* **2017**, 2752–2763.

[3] C. Knie, M. Utecht, F. Zhao, H. Kulla, S. Kovalenko, A. M. Brouwer, P. Saalfrank, S. Hecht, D. Bléger, *Chem. - A Eur. J.* **2014**, *20*, 16492–16501.

[4] F. N. Meng, Z. Y. Li, Y. L. Ying, S. C. Liu, J. Zhang, Y. T. Long, *Chem. Commun.* **2017**, *53*, 9462–9465.

[5] M. Varedian, V. Langer, J. Bergquist, A. Gogoll, *Tetrahedron Lett.* **2008**, *49*, 6033–6035.

[6] J. Bondebjerg, M. Grunnet, T. Jespersen, M. Meldal, *ChemBioChem* **2003**, *4*, 186–194.

[7] S. Wiedbrauk, H. Dube, *Tetrahedron Lett.* **2015**, *56*, 4266–4274.

[8] S. Kitzig, K. Rück-Braun, *J. Pept. Sci.* **2017**, *23*, 567–573.

[9] T. Cordes, D. Weinrich, S. Kempa, K. Riesselmann, S. Herre, C. Hoppmann, K. Rück-Braun, W. Zinth, *Chem. Phys. Lett.* **2006**, *428*, 167–173.

[10] O. Vázquez, O. Seitz, *Chem. Sci.* **2014**, *5*, 2850–2854.

[11] A. A. Beharry, O. Sadovski, G. A. Woolley, *J. Am. Chem. Soc.* **2011**, *133*, 19684–19687.

[12] T. Cordes, *Dissertation* **2008**.

[13] A. Savitzky, M. J. E. Golay, *Anal. Chem.* **1964**, *36*, 1627–1639.
